# The tapeworm interactome: inferring confidence scored protein-protein interactions from the proteome of *Hymenolepis microstoma*

**DOI:** 10.1186/s12864-020-6710-1

**Published:** 2020-05-07

**Authors:** Katherine James, Peter D. Olson

**Affiliations:** 1 Department of Applied Sciences, Northumbria University, Newcastle Upon Tyne, UK; 2 Department of Life Sciences, The Natural History Museum, Cromwell Road, London, UK

**Keywords:** Probabilistic network, Data integration, Interologs, Tapeworms, *Hymenolepis microstoma*

## Abstract

**Background:**

Reference genome and transcriptome assemblies of helminths have reached a level of completion whereby secondary analyses that rely on accurate gene estimation or syntenic relationships can be now conducted with a high level of confidence. Recent public release of the v.3 assembly of the mouse bile-duct tapeworm, *Hymenolepis microstoma*, provides chromosome-level characterisation of the genome and a stabilised set of protein coding gene models underpinned by bioinformatic and empirical data. However, interactome data have not been produced. Conserved protein-protein interactions in other organisms, termed interologs, can be used to transfer interactions between species, allowing systems-level analysis in non-model organisms.

**Results:**

Here, we describe a probabilistic, integrated network of interologs for the *H. microstoma* proteome, based on conserved protein interactions found in eukaryote model species. Almost a third of the 10,139 gene models in the v.3 assembly could be assigned interaction data and assessment of the resulting network indicates that topologically-important proteins are related to essential cellular pathways, and that the network clusters into biologically meaningful components. Moreover, network parameters are similar to those of single-species interaction networks that we constructed in the same way for *S. cerevisiae*, *C. elegans* and *H. sapiens*, demonstrating that information-rich, system-level analyses can be conducted even on species separated by a large phylogenetic distance from the major model organisms from which most protein interaction evidence is based. Using the interolog network, we then focused on sub-networks of interactions assigned to discrete suites of genes of interest, including signalling components and transcription factors, germline multipotency genes, and genes differentially-expressed between larval and adult worms. Results show not only an expected bias toward highly-conserved proteins, such as components of intracellular signal transduction, but in some cases predicted interactions with transcription factors that aid in identifying their target genes.

**Conclusions:**

With key helminth genomes now complete, systems-level analyses can provide an important predictive framework to guide basic and applied research on helminths and will become increasingly informative as new protein-protein interaction data accumulate.

## Background

Genomic resources for parasitic flatworms and other helminths have increased substantially over the last decade. Reference genomes of key species have undergone multiple iterations of improvement, employing new sequencing and algorithmic advances to produce more contiguous assemblies and reliable estimates of coding regions and other features [1]. At the same time, the diversity of helminth species with draft genomes continues to expand [2], enabling work on a broader range of species and more informative comparative analyses. Among flatworms, the human bloodfluke *Schistosoma mansoni* and the tapeworms *Echinococcus multilocularis* and *Hymenolepis microstoma* are now supported by near complete, chromosome-level assemblies [3–5], providing comprehensive and stable gene model estimates and syntenic relationships, as well as allowing the higher order architecture of their genomes to begin to be investigated. The unusually high level of completeness and quality of these assemblies makes them valuable not only for investigating these taxa, but also as models of the superphylum Lophotrochozoa which remains significantly under-represented in all areas of biological research.

*Hymenolepis microstoma*, the mouse bile-duct tapeworm, is one of three species of rodent/beetle-hosted hymenolepid tapeworms that have been used widely as laboratory models, as their entire life cycles can be passaged using hosts that are themselves model organisms [6]. A draft genome was published in 2013 [4] and was followed in 2015 by the public release of an up-dated assembly (v.2) based on additional Illumina data, as described in [5]. This assembly was used to investigate differentially-expressed genes among different life cycle stages and regions of the adult, strobilar worm [5], and for characterisation of the microRNA complement [7]. In 2018 long-read sequence and optical mapping data were added and all available genome data re-assembled, resulting in a complete assembly consisting of six scaffolds that correspond to their six haploid chromosomes [8]; any missing data that remain are likely to represent collapsed repeats rather than unique, non-repetitive sequence (Olson et al., in preparation). The 169 Mb v.3 assembly, including 10,139 gene models and an additional 1,290 splice variants, as well as RNA-seq data sets, is publicly available via WormBase ParaSite^1^ [9]. Thus, with the basic assembly and annotation of these inaugural helminth sequencing projects now effectively complete, we can begin to undertake systems-level analyses in parasitic flatworms for the first time.

Protein-protein interactions in cellular networks are known to be highly conserved [10, 11]. Evidence suggests that a simple set of rules characterizes all protein interaction networks [12], with network ‘hubs’ (highly-connected proteins) being conserved and essential [13–15], and having slower evolutionary rates [16] and significant sequence conservation [17]. Despite high-throughput interaction data having estimated false positive and negative rates as high as 90% and 50% [18], respectively, the conservation of hub proteins and their interactions remains detectable within eukaryotic species [19], and even between eukaryotes and prokaryotes [20]. Conserved interactions, termed ‘interologs’, can therefore be transferred between species [21–28], allowing systems-level analysis in organisms that lack empirical interaction data.

Here we produced a probabilistic, integrated network of interologs for *H. microstoma* using physical interaction data from sixteen different eukaryotic species obtained from the BioGRID database [29]. Probabilistic networks are more powerful than unweighted networks as they are annotated with a level of confidence in the evidence for each interaction by comparison with a benchmark ‘gold standard’ comprising a set of interactions believed, with high confidence, to be true interactions [30]. This benchmarking reduces noise from high-throughput data sets, produces consistent integration of interactions from different studies, and allows the use of thresholding and statistical algorithms that take these probabilities into account. We assessed the network by comparison of the major network parameters against networks of major model organisms produced using the same methods. We then used the network to identify highly-connected hub proteins, network clusters and interacting partners of genes of interest, including signalling components, transcription factors and germline multipotency genes, as well as genes differentially expressed between life stages. All data in our interaction network can be readily interrogated using Cytoscape [31], enabling users to explore predicted protein interactions for their own genes of interest. We expect that such network analyses will become an increasingly valuable resource for hypothesis generation, including predicting protein choke points for mitigating parasitism, determining processes shared between parasitic and free-living flatworms [32] and between flatworms and ourselves [22].

## Results

**Fig. 1:**
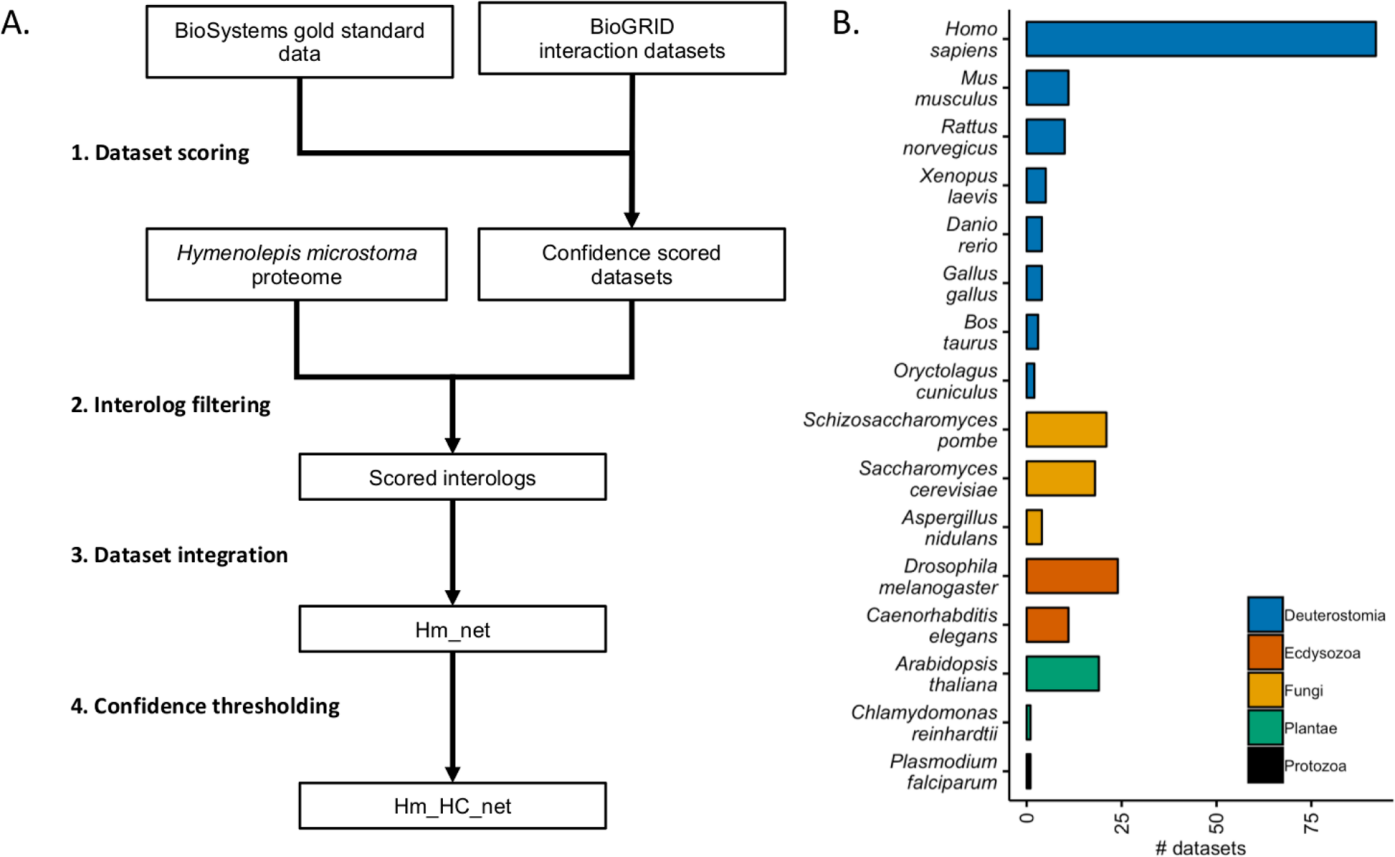
Network integration. **a** The *H. microstoma* interolog network was integrated using a four-stage scoring, integration, filtering and thresholding method. First, physical interaction from the BioGRID dataset were confidence scored against gold standard BioSystems data (1). These datasets were then filtered for interactions with Blast hits to the *H. microstoma* proteome (2) before integration using a weighted sum to produce the full interolog network, Hm_net (3). Finally, the network was thresholded based on interaction confidence to produce a high confidence network, Hm_HC_net (4). **b** Data from sixteen eukaryotic species were integrated in to Hm_net (classification based on [33])

**Fig. 2:**
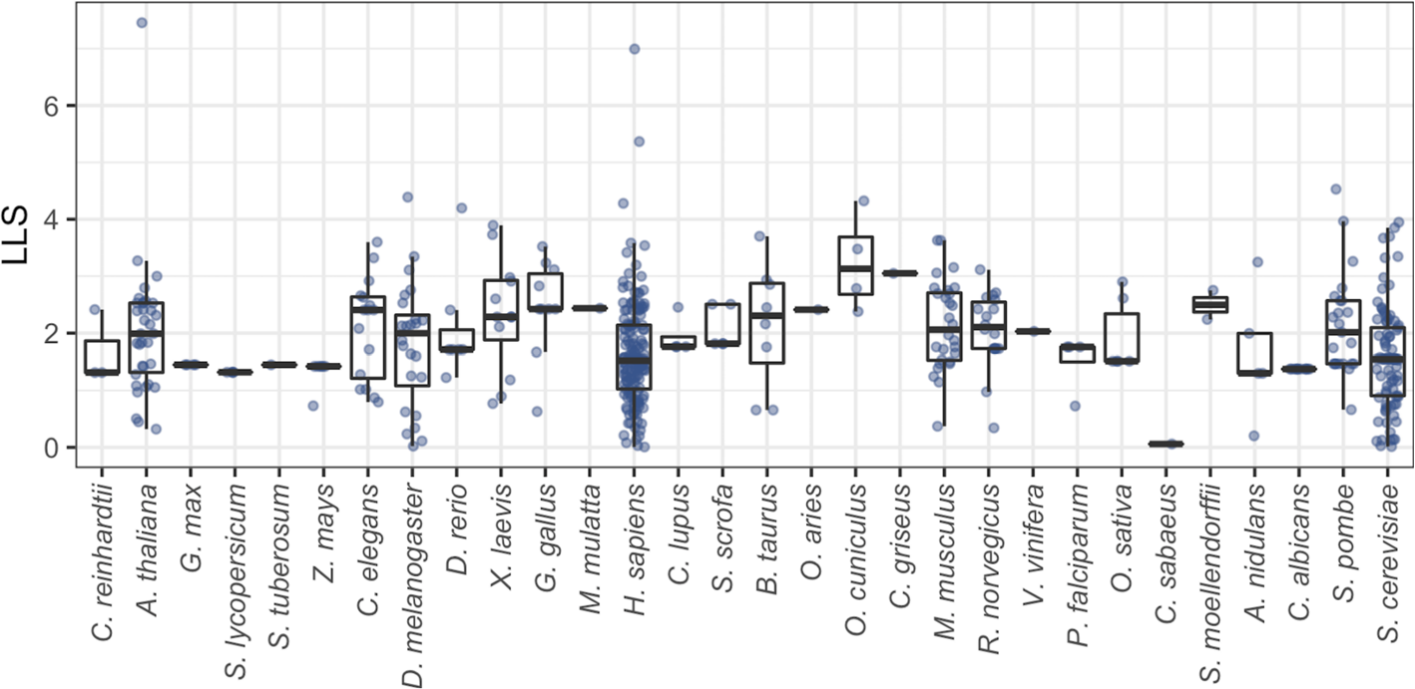
Dataset confidence scoring. The range of datasets loglikelihood (LLS) confidence scores for the BioGRID species. The majority of datasets were lost since they did not have any interactions that were interologs for *H. microstoma* gene models. Sixteen species were included in the final network (main text Table 1b)

**Fig. 3:**
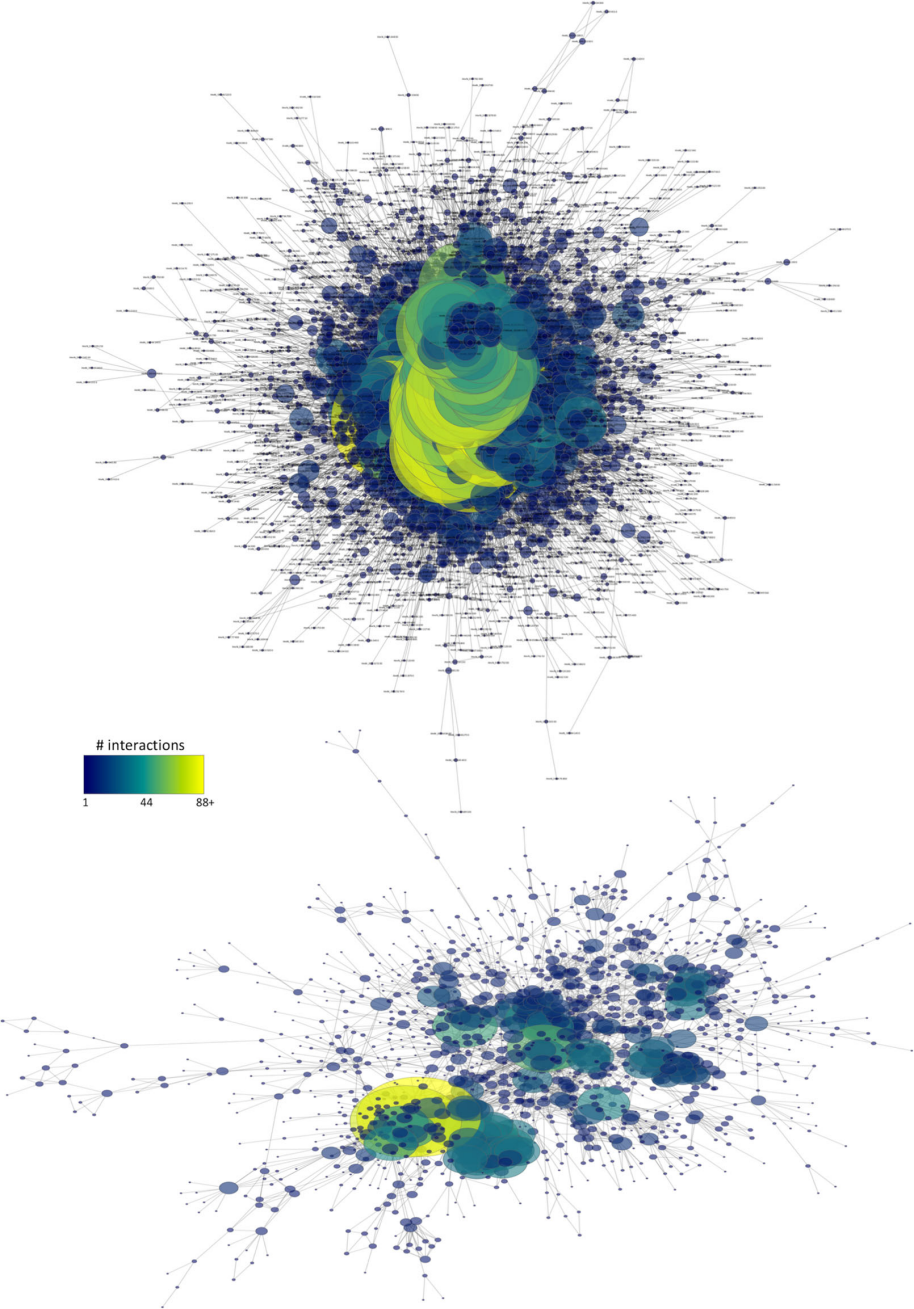
Graphical representation of Hm_net and Hm_HC_net. Hm_net (upper) comprising 3,408 proteins, ∼30% of the *H. microstoma* proteome, and 20,640 interactions (largest connected component is shown here). The largest component of Hm_HC_net (lower), comprising 1,260 proteins and 3,995 interactions with the highest confidence scores. In both cases protein nodes are coloured and sized by number of interactions. Two large hub proteins (yellow) remain following the thresholding

**Fig. 4:**
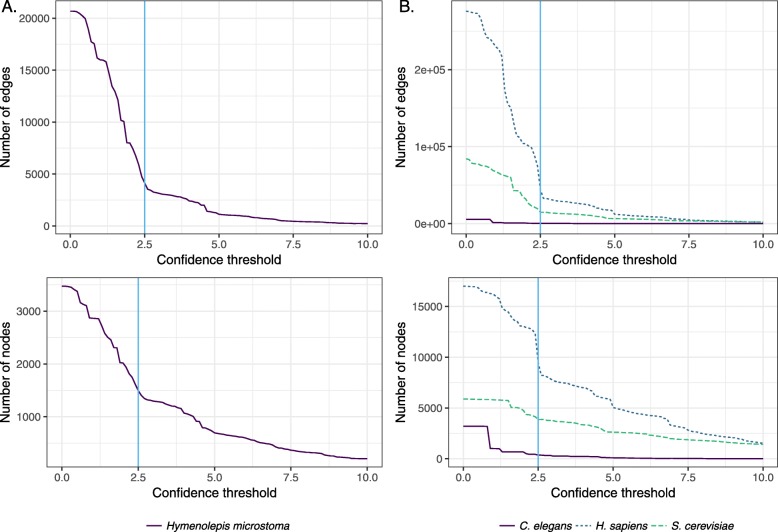
Confidence thresholds. **a** Hm_net was filtered at a confidence score of 2.5 (vertical blue line, upper: number of edges, lower: number of nodes), corresponding to a drop in distribution of confidence scores, to produce the high confidence Hm_HC_net. **b** The *H. sapiens* and *S. cerevisiae* networks have a similar drop in confidence score distribution (upper: number of edges, lower: number of nodes) at a score of 2.5 despite being larger and far more densely-connected than Hm_net. The majority of *C. elegans* interaction scores are <1.0

We integrated a probabilistic network of *H. microstoma* interologs using a four-step scoring, filtering, integration and thresholding pipeline (Fig. 1a; Methods). BioGRID datasets were first filtered to remove bacterial data before confidence scoring against a gold standard data set derived from the BioSystems database. A total of 528 datasets were produced (Additional file 1, Tables S1 and S2), 428 of which had a positive confidence score (Fig. 2). Blastx was then used to identify proteins from the BioGRID species which had significant similarity to those of the *H. microstoma* proteome. Finally, the Blast hits were mapped to the scored datasets and the dataset confidence scores integrated using a weighted sum (Methods). In total, 230 data sets from 16 species were included in the final integration step (Fig. 1b), resulting in a network of 3,474 proteins (∼30*%* of the *H. microstoma* somatic proteome) and 20,684 interactions: Hm_net (Fig. 3, upper). The network scores were also filtered using a threshold to produce a high confidence sub-network of 1,494 proteins and 4139 interactions with the highest weighted evidence: Hm_HC_net (Fig. 3, lower and Fig. 4a). The full tapeworm interolog network, Hm_net, high confidence sub-network, Hm_HC_net and network annotations are provided in Additional files 2, 3 and 4 in a tab-delimited format suitable for use with Cytoscape and other network analysis software.

**Table 1:** Network topology

	***H. sapiens***	***S. cerevisiae***	***C. elegans***	**Hm_net**
Protein (all)	17001	5883	3194	3474
Interactions (all)	276002	84277	5572	20684
Connected components	11	1	96	26
Proteins (LC)	16980	5883	2969	3408
Interactions (LC)	275991	84277	5442	20640
Proteome coverage (%)*	56.5	89.5	14.7	33.6
Largest hub*	TRIM25	NAB2	gei-4	Cullin 3
Clustering coefficient*	0.108	0.275	0.036	0.126
Diameter*	9	6	13	10
Characteristic path length*	3.082	2.483	4.802	3.837
Mean number of interactions*	32.508	28.651	3.666	12.113

The network and topological statistics of the of Hm_net in comparison to single species networks. Topological statistics (*) are calculated for the largest component (LC) only

We assessed the network using a variety of network analysis techniques. Initially, we compared the network to protein-protein interaction networks from three model species: human, yeast, and *C. elegans* to determine how closely the topology of Hm_net resembles networks produced directly from experimental protein-protein interaction data. We then investigated the topologically-important proteins and network clusters of Hm_HC_net in an exploratory manner. Finally, we used the network to ask whether it could predict interaction partners for groups of genes relating to development and to genes differentially expressed between larval and adult worms, as enumerated above.

### Hm_net is topologically comparable to protein-protein interaction networks from major model organisms

Network topological parameters are often used to characterise the global properties of biological networks [34]. We compared the topology of the *H. microstoma* interolog network to those of humans, yeast and *C. elegans*, integrated using the same probabilistic methodology, in order to asses how well Hm_net resembles a real protein-protein interaction network derived from a single species. The human network consisted of 153 data sets (16 low-throughput (LTP)), yeast 89 data sets (17 LTP) and *C. elegans* 16 data sets (14 LTP). The *H. sapiens* and *S. cerevisae* networks had a similar confidence score distribution to Hm_net with a large proportion of interactions scoring below 2.5, while the majority of *C. elegans* interactions scored below 1.0 (Fig. 4b).

The human network was by far the largest of the four networks, reflecting the larger proteome and multiple tissue types (Table 1). In contrast, the yeast network was the most dense having the smallest diameter (i.e. minimum number of links that separate the two most distant proteins in a network) and a single connected component of interactions, reflecting its single cell type as well as a larger number of high-throughput data sets. Although containing fewer proteins, Hm_net is similar to the other networks in terms of its overall topology. Hm_net, has the highest clustering coefficient of the four, likely due to the multiple sources of interolog evidence resulting in denser connectivity between related proteins. By contrast, the *C. elegans* network is smaller and more dispersed with a larger diameter and characteristic path length.

The protein with the largest number of interactions in Hm_net was Cullin3 (n = 257), a protein involved in ubiquitination that has source interactions from ten eukaryotic species. The proteins with the largest number of interactions in the other species were the immune response E3 ubiquitin ligase TRIM25 in *H. sapiens* (*n* = 2384), the NAB2 mRNA binding protein in *S. cerevisiae* (*n*= 2580) and gei-4, a signal transduction protein, in *C. elegans* (*n* = 181). TRIM25 and NAB2 both have a large number of BioGRID interactions, 2,593 and 2,689, respectively, very few of which were lost during the scoring, mapping and integration process. In contrast, gei-4 has just 181 interactions, all of which are present in the final network.

**Fig. 5:**
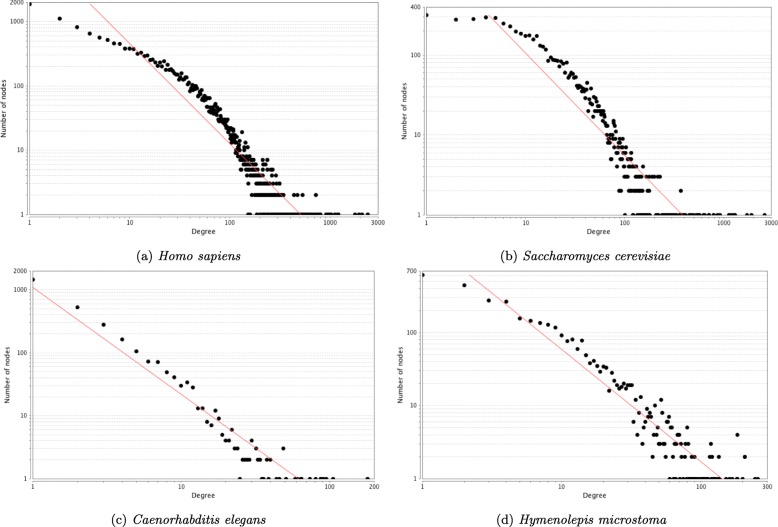
Degree distribution. The degree distribution of the four networks with the power law fitted (red). In each case the distribution is a good fit for the power law, indicating that the network has a small number of highly-interacting ‘hub’ proteins, which is a hallmark of protein-protein interaction networks: **a** correlation 0.851, R-squared 0.878; **b** correlation 0.660, R-squared 0.827; **c** correlation 0.998, R-squared 0.902; **d** correlation 0.896, R-squared 0.877

**Fig. 6:**
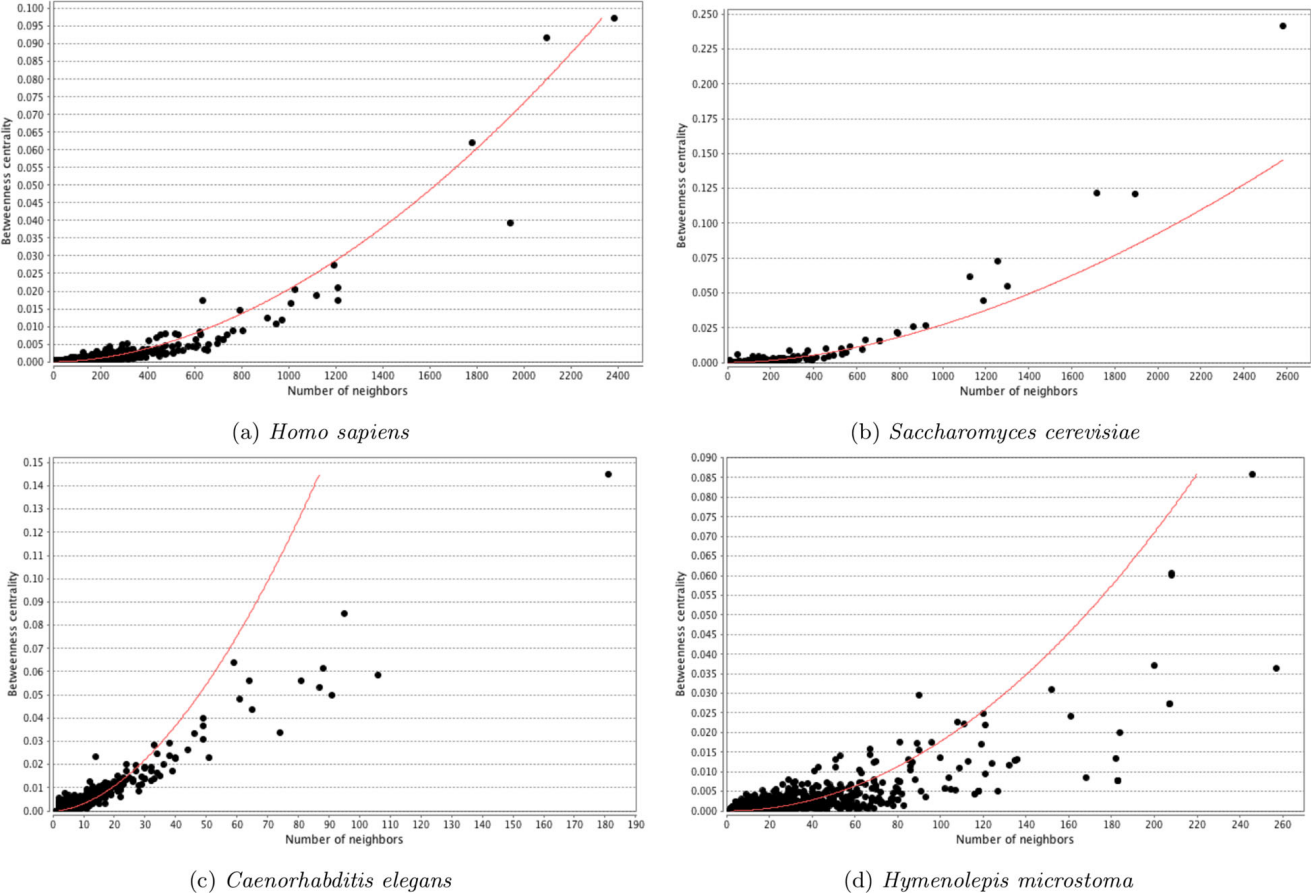
Betweenness centrality distribution. The distribution of betweenness centrality of the four networks with the power law fitted (red). In each case the distribution is a good fit for the power law, indicating that the network has a small number of high betweenness proteins, with the majority being high betweenness. **a***Homo sapiens*. **b***Saccharomyces cerevisiae*. **c***Caenorhabtitis elegans*. **d***Hymenolepis microstoma*

**Fig. 7:**
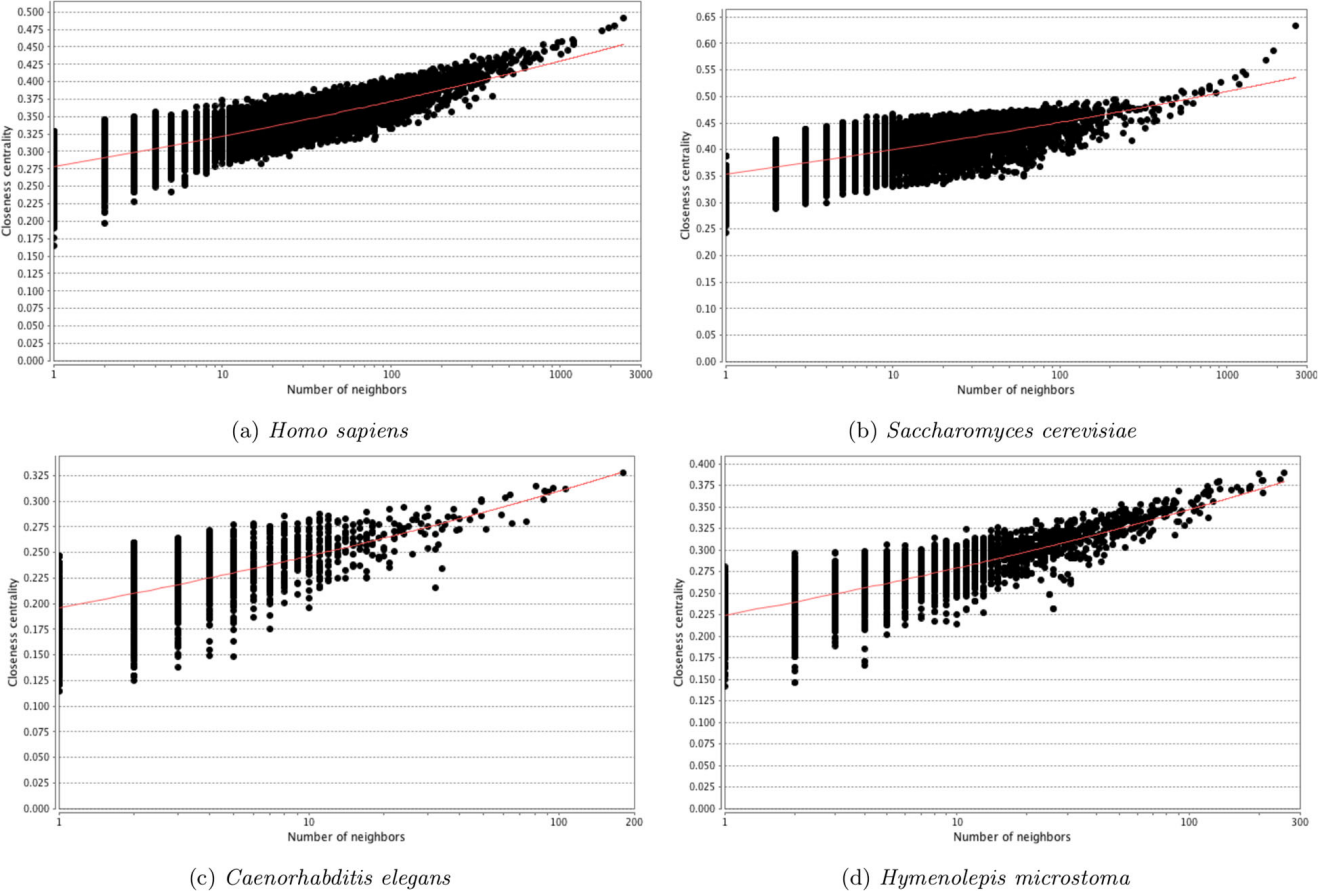
Closeness centrality distribution. The distribution of closeness centrality of the four networks with the power law fitted (red). In each case the distribution is a good fit for the power law, indicating that the network has a small number of high closeness proteins, with the majority being high closeness. **a***Homo sapiens*. **b***Saccharomyces cerevisiae*. **c***Caenorhabtitis elegans*. **d***Hymenolepis microstoma*

**Fig. 8:**
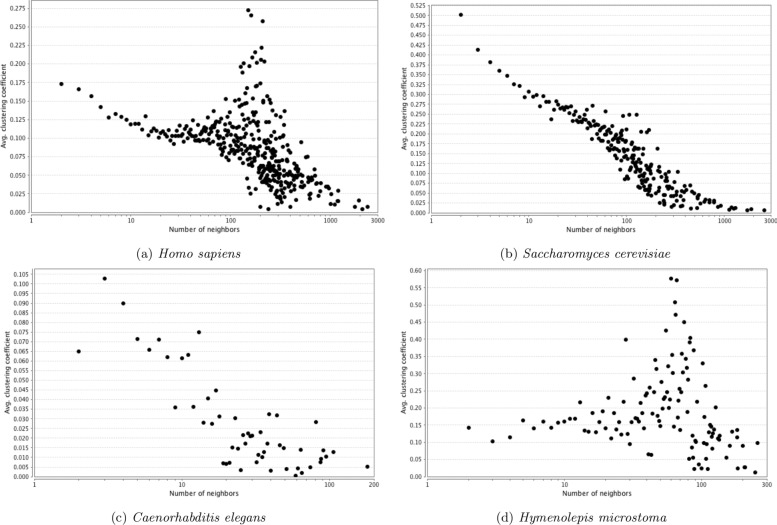
Clustering coefficient distribution. The distribution of clustering coefficient of the four networks. In general, the proteins with a higher number of neighbours have a higher clustering co-efficient in all four cases. The distribution of Hm_net is most similar to that of *H. sapiens*, in which a group of proteins with a lower number of neighbours form a high clustering co-efficient peak, likely reflecting the larger proportion of human data contributing to the interologs. **a***Homo sapiens*. **b***Saccharomyces cerevisiae*. **c***Caenorhabtitis elegans*. **d***Hymenolepis microstoma*

The distribution of topological parameters in all four networks were similar, with the scale of the scores reflecting the size and density of the networks (Figs. 5, 6, 7 and 8). The degree (i.e. number of protein interaction) distribution of Hm_net (Fig. 5a) indicates that the network exhibits scale-free behaviour [35] in that it has a small number of highly connected proteins with the distribution obeying the power law, which is a hallmark of protein-protein interaction networks [14, 36]. The other three networks also have scale-free behaviour, although *S. cerevisiae* to a lesser extent (Fig. 5b-d). The distribution patterns of betweenness and closeness centrality [37] (measures of a protein’s topological importance) were also similar in all four networks (Figs. 6 and 7), whereas the clustering coefficient [38] (i.e. degree of connectivity in a protein’s immediate neighborhood) distribution of Hm_net was more similar to that of *H. sapiens*, reflecting the larger proportion of human data contributing to the interologs (Fig. 8).

### Toplogically-important proteins of the high confidence network (Hm_HC_net) are involved in essential cellular processes

The topological statistics of a network may be used to identify the most important proteins in the network. We chose to assess the topologically-important proteins of the largest connected component of Hm_HC_net (1260 proteins and 3995 interactions) based on three topological scores produced by NetworkAnalyser:

1. Protein degree (number of interaction partners) to identify the top network ‘hubs’ (highly interacting proteins) [13].
2. Betweenness centrality (BC) to identify proteins that lie between dense areas of the network [37].
3. Closeness centrality (CC) to identify the most central proteins in the network in terms of information flow [37].

Network hubs in protein interaction networks are often conserved and essential proteins [13–15]. The largest network hub with 88 interactions, HmN_000772200, is a cell cycle division 5-like (CDC5L) protein that is involved in the G2/M transition and known to be required for pre-mRNA splicing. The second largest hub, HmN_000015300, was also a putitive pre-mRNA processing factor. Of the remaining top network hubs, a large number of the proteins were involved in gene expression; eight ribosomal proteins; two translation initiation factors; one RNA polymerase subunit, and a histone deacetylase (Table 2). Five of the hubs were cullin family proteins which play an intrinsic role in post-translational modification of protein via ubiquitination [39]. Four of these cullin proteins, (HmN_000063500, HmN_000629900, HmN_003003610 and HmN_003003620), have interactions resulting from the same interolog evidence, resulting in identical interactions and topological statistics in the network. The remaining top hubs are two small nuclear ribonucleoproteins, a heat shock protein that is involved in protein folding, two protein kinases, and microfibrillar associated protein 1 also annotated to pre-mRNA splicing.

**Table 2:** Network hubs

**Gene model**	**Degree**	**BC**	**CC**	**Description**
HmN_000772200	88	0.131	0.284	Cell division cycle 5 protein
HmN_000015300	79	0.101	0.283	Pre-mRNA processing factor 19
HmN_000799800	52	0.129	0.297	Heat shock protein 90 alpha
HmN_003006230	46	0.135	0.290	DNA-directed RNA polymerase subunit RBP1
HmN_000008200	42	0.046	0.262	Cullin 3
HmN_000077700	41	0.007	0.231	40S ribosomal protein S3a
HmN_000217500	41	0.004	0.234	40S ribosomal protein S2
HmN_002028700	39	0.006	0.247	Probable U2 small nuclear ribonucleoprotein A’
HmN_003003820	39	0.034	0.268	Eukaryotic initiation factor 4A-III
HmN_003003830	39	0.034	0.268	Eukaryotic initiation factor 4A-III
HmN_000704800	38	0.135	0.315	Cyclin-dependent kinase 2
HmN_000755100	38	0.019	0.245	Microfibrillar associated protein 1
HmN_000632800	38	0.004	0.243	116 kDa U5 small nuclear ribonucleoprotein component
HmN_000018400	37	0.069	0.255	Histone deacetylase 1
HmN_000932000	37	0.006	0.232	40S ribosomal protein S16
HmN_000002900	37	0.001	0.223	40S ribosomal protein S4
HmN_003026960	36	0.002	0.231	40S ribosomal protein S8
HmN_000899300	35	0.001	0.222	40S ribosomal protein S13
HmN_000632900	35	0.001	0.222	40S ribosomal protein S23
HmN_000063500	34	0.014	0.269	Cullin 1
HmN_000629900	34	0.014	0.269	Cullin 1
HmN_003003610	34	0.014	0.269	Cullin 1
HmN_003003620	34	0.014	0.269	Cullin 1
HmN_000144900	33	0.068	0.302	Cyclin-dependent kinase 2
HmN_000490400	33	0.001	0.231	60S ribosomal protein L4

The top twenty-five network hubs ranked by degree (number of interactions). BC: betweenness centrality; CC: closeness centrality

Centrality statistics measure a protein’s importance to information flow through the network [37]. Betweenness centrality is a measure of the amount of influence a protein has on information flow based on the number of shortest paths between protein pairs on which it lies. High scoring proteins that also have low degree (few interactions), termed ‘bottlenecks’, are often highly conserved and essential [40, 41]. Closeness centrality also measures information flow through a protein based on how short the shortest paths are from that protein to all other proteins in the network. A high score indicates the ability to communicate with other network members through a small number of intermediaries and has been used to identify key components of metabolic pathways [42].

Of the top scoring proteins for betweenness centrality (Table 3) ten are also network hubs. The other fifteen proteins are six involved in the cell cycle and replication, three cytoskeletal, two histone-related, two ubiquitination, one clarthrin chain and one proteasomal protein. Four of the cell cycle proteins are transitional endoplasmic reticulum ATPases (HmN_000846600, HmN_003022520 and HmN_003022580) have interactions resulting from the same interolog evidence and, therefore, identical interactions and topological statistics in the network. The majority of high closeness centrality proteins (20 of 25) are hubs, high betweenness or both (Table 4). The remaining high CC are a splicing factor, a chaperonin, a SNW domain containing transcriptional protein, and two proteins involved in the cell cycle and replication.

**Table 3:** Betweenness centrality

**Gene model**	**Degree**	**BC**	**CC**	**Description**
HmN_003006230	46*	0.135	0.290	DNA-directed RNA polymerase subunit RBP1
HmN_000704800	38*	0.135	0.315	Cyclin-dependent kinase 2
HmN_000772200	88*	0.131	0.284	Cell division cycle 5 protein
HmN_000799800	52*	0.129	0.297	Heat shock protein 71 kDa protein 90 alpha
HmN_000015300	79*	0.101	0.283	Pre-mRNA processing factor 19
HmN_003048860	24	0.097	0.277	Actin
HmN_003006810	28	0.074	0.281	Histone acetyltransferase p300
HmN_000018400	37*	0.069	0.255	Histone deacetylase 1
HmN_000144900	33*	0.068	0.302	Cyclin-dependent kinase 2
HmN_002231000	32	0.051	0.260	26S proteasome non-ATPase regulatory subunit 2
HmN_000536300	24	0.047	0.265	Tubulin gamma chain-1
HmN_000547600	12	0.047	0.267	Histone H2B
HmN_000008200	42*	0.046	0.262	Cullin 3
HmN_000237800	21	0.043	0.254	Dual specificity protein phosphatase cdc14a
HmN_000405000	14	0.038	0.258	Clathrin heavy chain 1
HmN_000846600	30	0.037	0.288	Transitional endoplasmic reticulum ATPase
HmN_003022520	30	0.037	0.288	Transitional endoplasmic reticulum ATPase
HmN_003022580	30	0.037	0.288	Transitional endoplasmic reticulum ATPase
HmN_002012300	10	0.036	0.241	Ubiquitin-conjugating enzyme E2 variant 3
HmN_003003820	39*	0.034	0.268	Eukaryotic initiation factor 4A-III
HmN_003003830	39*	0.034	0.268	Eukaryotic initiation factor 4A-III
HmN_000066300	23	0.033	0.259	E3 ubiquitin ligase RING3
HmN_003005250	9	0.033	0.234	Dynein heavy chain
HmN_000150200	23	0.031	0.256	Proliferating cell nuclear antigen
HmN_000372000	17	0.030	0.271	DNA replication licensing factor MCM2

The top twenty-five protein ranked by betweenness centrality. Proteins that are also top 20 protein hubs (Table 2) are denoted with *. BC: betweenness centrality; CC: closeness centrality

**Table 4:** Closeness centrality

**Gene model**	**Degree**	**BC**	**CC**	**Description**
HmN_000704800	38*	0.135**	0.315	Cyclin-dependent kinase 2
HmN_000144900	33*	0.068**	0.302	Cyclin-dependent kinase 2
HmN_000799800	52*	0.129**	0.297	Heat shock protein 71 kDa protein 90 alpha
HmN_003006230	46*	0.135**	0.290	DNA-directed RNA polymerase subunit RBP1
HmN_000846600	30	0.037**	0.288	Transitional endoplasmic reticulum ATPase
HmN_003022520	30	0.037**	0.288	Transitional endoplasmic reticulum ATPase
HmN_003022580	30	0.037**	0.288	Transitional endoplasmic reticulum ATPase
HmN_000772200	88*	0.131**	0.284	Cell division cycle 5 protein
HmN_000015300	79*	0.101**	0.283	Pre mRNA processing factor 19
HmN_003006810	28	0.074**	0.281	Histone acetyltransferase p300
HmN_003048860	24	0.097**	0.277	Actin
HmN_000372000	17	0.030**	0.271	DNA replication licensing factor MCM2
HmN_000063500	34*	0.014	0.269	Cullin 1
HmN_000629900	34*	0.014	0.269	Cullin 1
HmN_003003610	34*	0.014	0.269	Cullin 1
HmN_003003620	34*	0.014	0.269	Cullin 1
HmN_003003820	39*	0.034**	0.268	Eukaryotic initiation factor 4A-III
HmN_003003830	39*	0.034**	0.268	Eukaryotic initiation factor 4A-III
HmN_000547600	12	0.047**	0.267	Tubulin gamma chain-1
HmN_000536300	24	0.047**	0.265	Histone H2B
HmN_000476900	23	0.025	0.265	Splicing factor 3b subunit 3
HmN_000109300	14	0.011	0.264	Cyclin dependent kinase 7
HmN_000744000	16	0.025	0.263	Chaperonin containing TCP1 subunit 2 (beta)
HmN_000541900	13	0.018	0.262	Replication protein A 70 kDa DNA-binding subunit
HmN_000463400	22	0.028	0.262	SNW domain containing protein 1

The top twenty-five protein ranked by closeness centrality. Proteins that are also top 20 protein hubs (Table 2) are denoted with * and top 20 betweenness centrality (Table 3) with **. BC: betweenness centrality; CC: closeness centrality

### Network clusters of the high confidence network (Hm_HC_net) correspond to biological modules and processes

We used the MCODE algorithm to identify tightly connected areas of the largest component of Hm_HC_net since clusters in protein-protein networks from model species generally correspond to complexes of proteins involved in the same biological process [43]. A total of 38 clusters were identified in Hm_net, ranging from MCODE score 26.5 to 2.7, and 3 to 27 proteins in size.

**Fig. 9:**
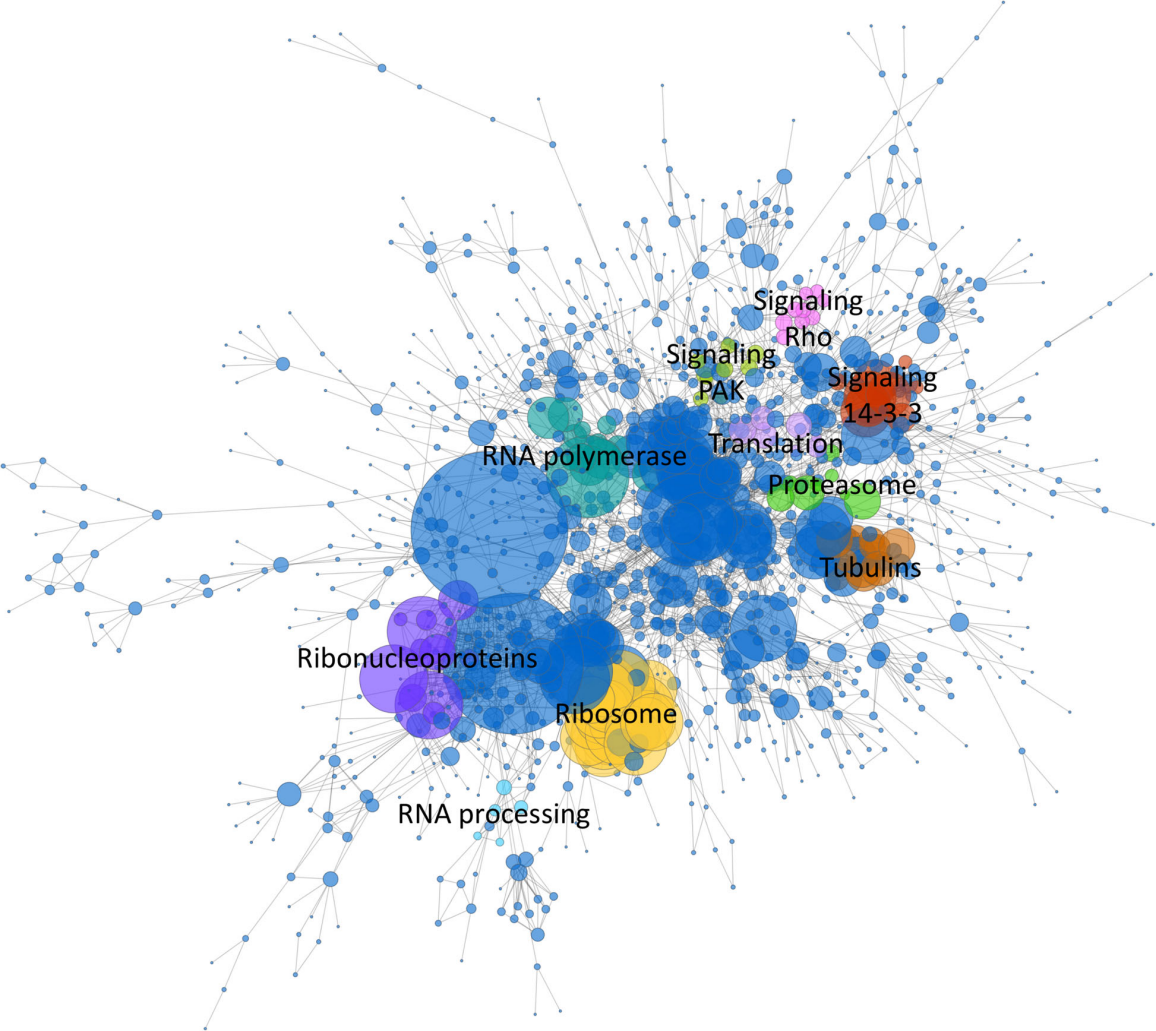
Clustered network. The ten highest scoring MCODE clusters are highlighted in Hm_net. Protein nodes are sized by number of interactions. Cluster 1: yellow, cluster 2: turquoise, cluster 3: green, cluster 4: purple, cluster 5: brown, cluster 6: mauve, cluster 7: light blue, cluster 8: red, cluster 9: pale green, cluster 10: pink. Full cluster annotations are provided in Additional file 1 Table S1

**Fig. 10:**
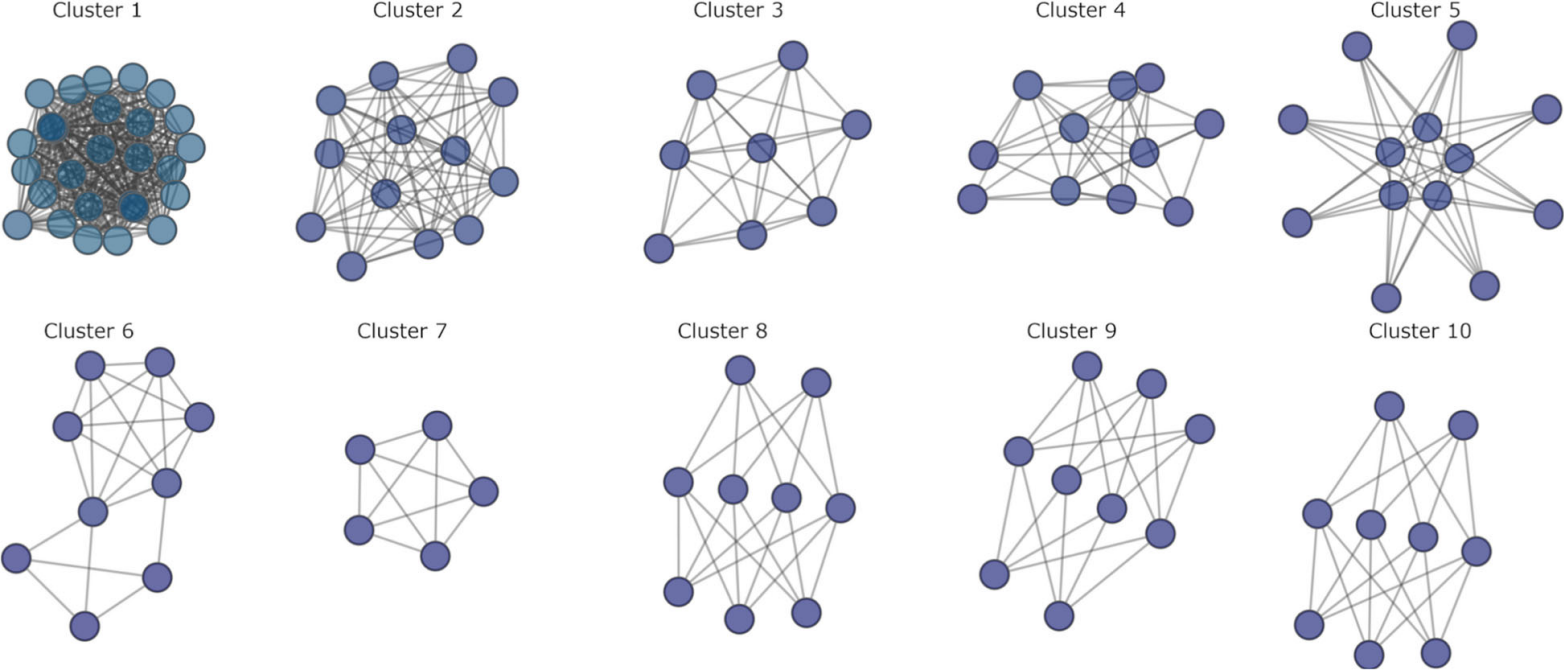
Network clusters. The ten largest network clusters (Tables S4, main text Table 5 and Fig. 4): cluster 1 - ribosome; cluster 2 - DNA-dependent RNA polymerase; cluster 3 - proteasome; cluster 4 - spliceosome; cluster 5 - tubulins; cluster 6 - translation initiation complex; cluster 7 - snRNP binding; cluster 8 - 14-3-3 signaling; cluster 9 - PAK kinases; cluster 10 - RhoA

The ten highest scoring clusters represent proteins with related biological functions (Figs. 9 and 10; Table 5). The largest and highest scoring cluster comprises 27 proteins, 26 of which are ribosomal subunits in addition to a single proteasome subunit, HmN_000306800. Eight of the ribosomal proteins of cluster 1 are network hubs. Clusters 2 and 3 represent groups DNA-directed RNAP and proteasome proteins, respectively, with the exception of HmN_003000770 in cluster 3, which is a SWI/SNF-related chromatin regulator. A single RNA polymerase II subunit of cluster 2, HmN_003006230, is a network hub. Cluster 4 contains 11 proteins related to mRNA processing and the spliceosome, 3 of which are hub proteins. Cluster 5 contains proteins that are mainly related to microtubules, 8 tubulins, dynactin and dystonin, in addition to an ADP-ribosylation factor and two unannotated proteins, HmN_000742700 and HmN_000742800.

**Table 5:** Network clusters

**Cluster**	**MCODE Score**	**Proteins**	**Interactions**	**Description**
1	26.462	27	344	Ribosome
2	11.667	13	70	DNA-dependent RNA polymerase
3	7.429	8	26	Proeasome
4	7.200	11	36	Spliceosome
5	6.667	13	40	Tubulins
6	5.250	9	21	Translation initiation complex
7	5.000	5	10	snRNP binding (LSm)
8	5.000	9	20	14-3-3 proteins
9	5.000	9	20	PAK kinases
10	5.000	9	20	RhoA

Cluster metrics for the ten clusters shown in Fig. 9. Cluster descriptions are based on the majority of annotations to the cluster proteins; full cluster annotations are provided in Additional file 1 Table S1

Cluster 6 comprises six eukaryotic translation initiation factors and two subunits of the COP9 signalosome, a regulator of the Cullin-RING proteins. Cluster 7 contains four of the Lsm proteins which bind U6 snRNPs and a probable snRNP. Finally, clusters 8, 9 and 10 are signalling and regulatory clusters each comprising several kinases in addition to members of the 14-3-3 proteins, PAK kinases and RhoA pathways, respectively. Full cluster annotations, including hub proteins, are provided in Additional file 1 Table S1.

### Specific genes of interest: signalling, transcription and germline-related genes

**Fig. 11:**
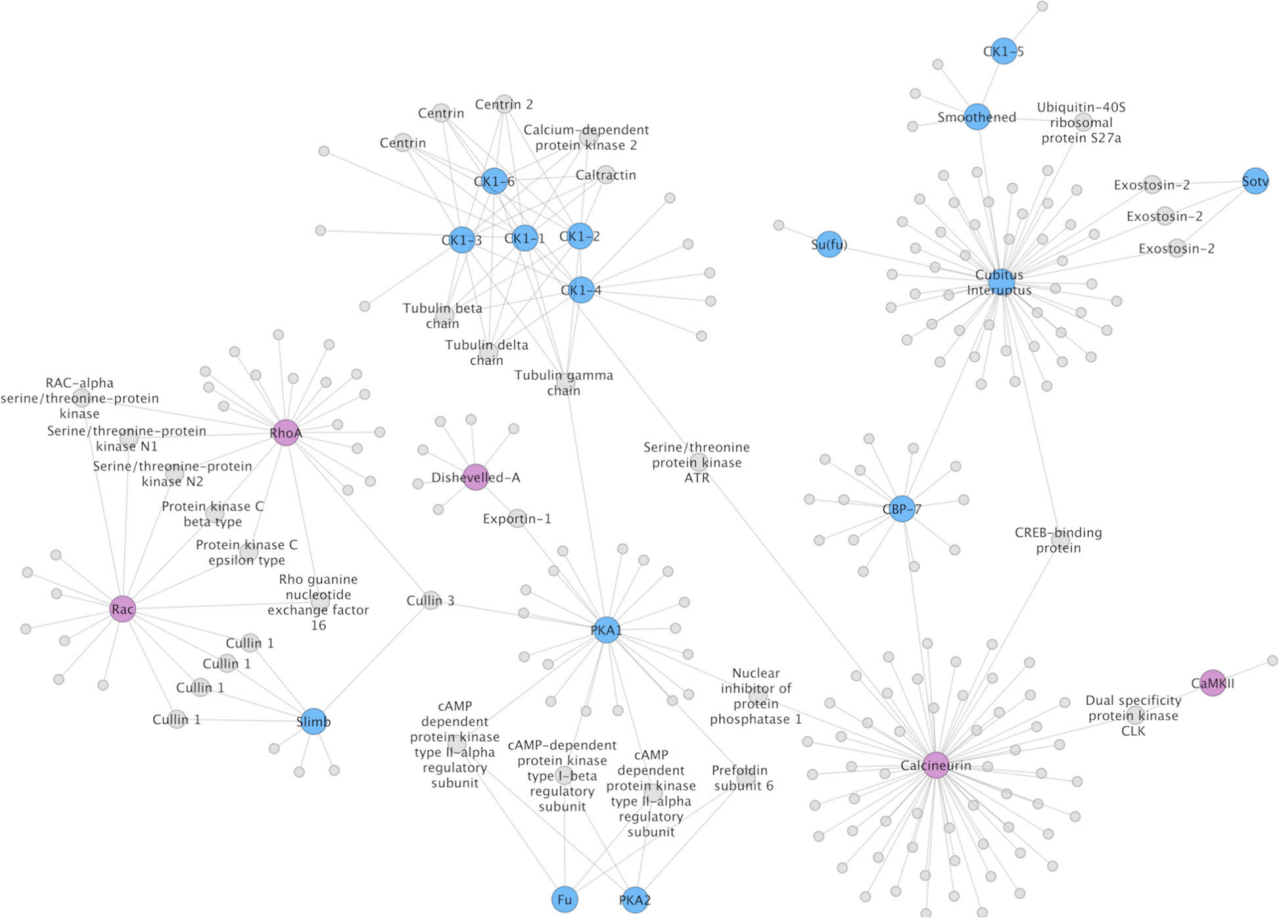
Hedgehog and Wnt signaling components. Predicted interactions within and between intracellular transducers of Hedgehog (blue) and Wnt (purple) signaling pathways

**Fig. 12:**
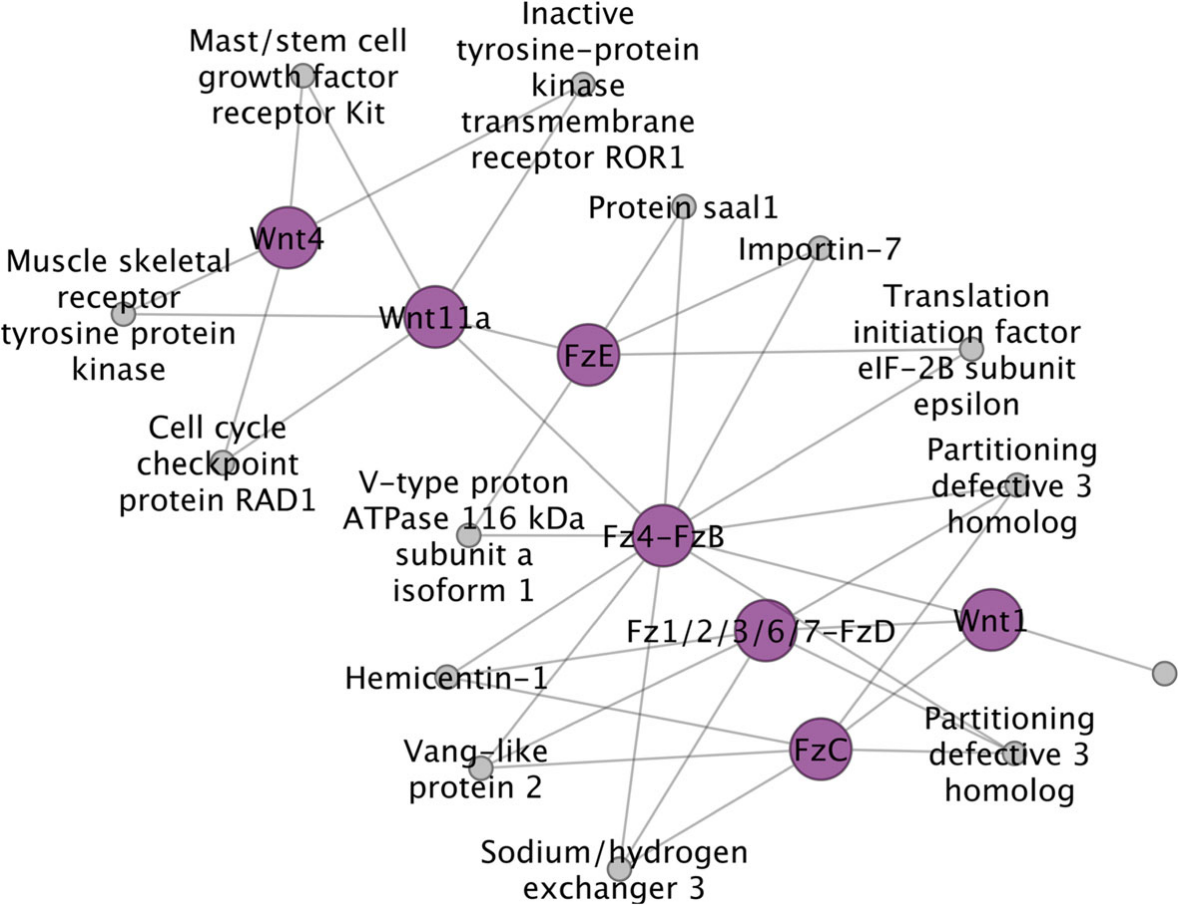
Wnt ligands and frizzled transmembrane receptors. Predicted interactions between the primary effectors of Wnt signaling

**Fig. 13:**
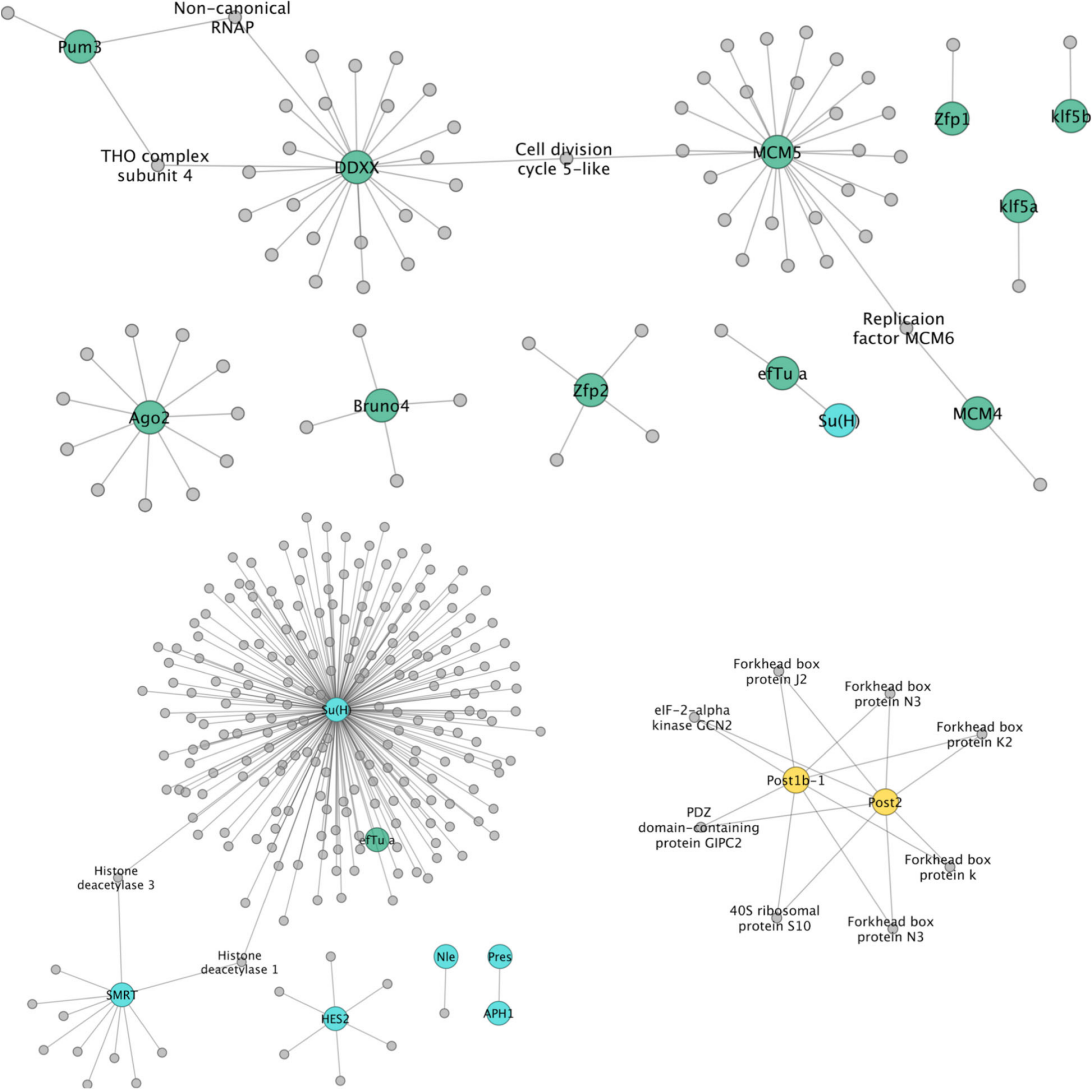
Germline ‘multipotency’ genes, Notch signaling components and Hox genes. Predicted interactions of putative multipotency proteins (teal), Notch signaling components (blue) and two posterior Hox transcription factors (yellow)

Probably the most common use of interactome prediction is to identify proteins that, based on knowledge in other organisms, are likely to be associated with specific genes of interest (GOI). To this end, we examined specific suites of proteins representing components of select signalling pathways, transcription factors and germline/stem-cell-related multipotency genes [4, 5, 44]. These suites of genes were chosen because of an interest in the developmental genetics of these organisms [5], and because they have been hand-curated, making their IDs more reliable than the gene models identified solely by automated means. The results of these are shown in Figs. 11, 12 and 13. As expected, the majority of the GOIs that were present in the network, and that also showed the largest number of interactions, were signal transduction components or cell cycle regulators that are highly conserved and among the former, often operate across multiple pathways. This is illustrated in Fig. 11 in which intracellular components of Wnt and Hedgehog signalling are predicted to be connected functionally by way of cullin proteins (which help direct ubiquitin-mediated protein destruction), protein kinases (involved in phosphorylation), and additional factors. Among these are some of the most highly connected proteins among the GOIs, such as RhoA, a hydrolase that acts in the Wnt planar cell polarity pathway and Calcineurin, a protein phosphatase involved in dephosphorylation which acts in the calcium-dependent Wnt pathway (Fig. 11). Additional examples are Cubitus interruptus, a zinc finger transcription factor responsible for activating downstream target genes (such as Wnt1) in Hedgehog signalling, and Suppressor of hairy, a bi-functional protein that mediates activation or repression of other proteins in the Notch signalling pathway (Fig. 13).

Figure 12 shows interactions between and within Wnt ligands and their canonical receptors, the frizzled transmembrane proteins. Results predict direct interactions between the posterior morphogen Wnt1 and three of the five frizzled receptors in their genome (Fz4, Fz1/2/3/6/7 and FzC) [45]. The posterior Wnt11 ortholog Wnt11a is also predicted to interact with Fz4, but also with FzE (see Additional file 1, Table S3 for corresponding gene models). This is consistent with the posterior expression of Wnt11’s and Fz4 during larval metamorphosis of tapeworms [46], as well as during regenerative growth in planarians [47]. Wnt4, by contrast, is not linked to a frizzled protein. Among interactions with other proteins, links between Wnt4 and Wnt11a to the cell cycle checkpoint protein Rad1 may be one means by which the canonical, *β*-catenin-dependent, Wnt pathway can regulate cell proliferation and thus growth.

Figure 13 shows germline, or stem-cell, ‘multipotency’ proteins, components of the Notch signalling pathway (discussed above) and Hox transcription factors found in Hm_net. Relatively few putative stem-cell related proteins were found in the network, but those that were are connected, as expected, by other regulators of the cell cycle such as CDC5-like. Similarly, most predicted interactions are with housekeeping or cell cycle regulatory genes. However, one of two zinc finger transcription factors putatively associated with flatworm stem cells [48] has four predicted interacting proteins, all of which are SMAD factors, the intracellular transducers of the TGF *β*/BMP signalling pathways.

Only two Hox family transcription factors were found to be present in the network: a bona fide ‘posterior’ Post2/AbdB ortholog and one of multiple Post1/Post2-like paralogs [32]. Although the sequences of these two proteins are very divergent, both are annotated with identical interacting proteins, and thus would be predicted to be playing the same role in the organism. Interestingly, five of the eight associated proteins are forkhead box (FOX) transcription factors, which are known to interact with Hox genes [49], and the network results thus provide predictions of which of the numerous FOX proteins in tapeworms to investigate in relation to Hox expression.

### Differentially expressed genes: comparing the interactomes of larval and adult worms

**Fig. 14:**
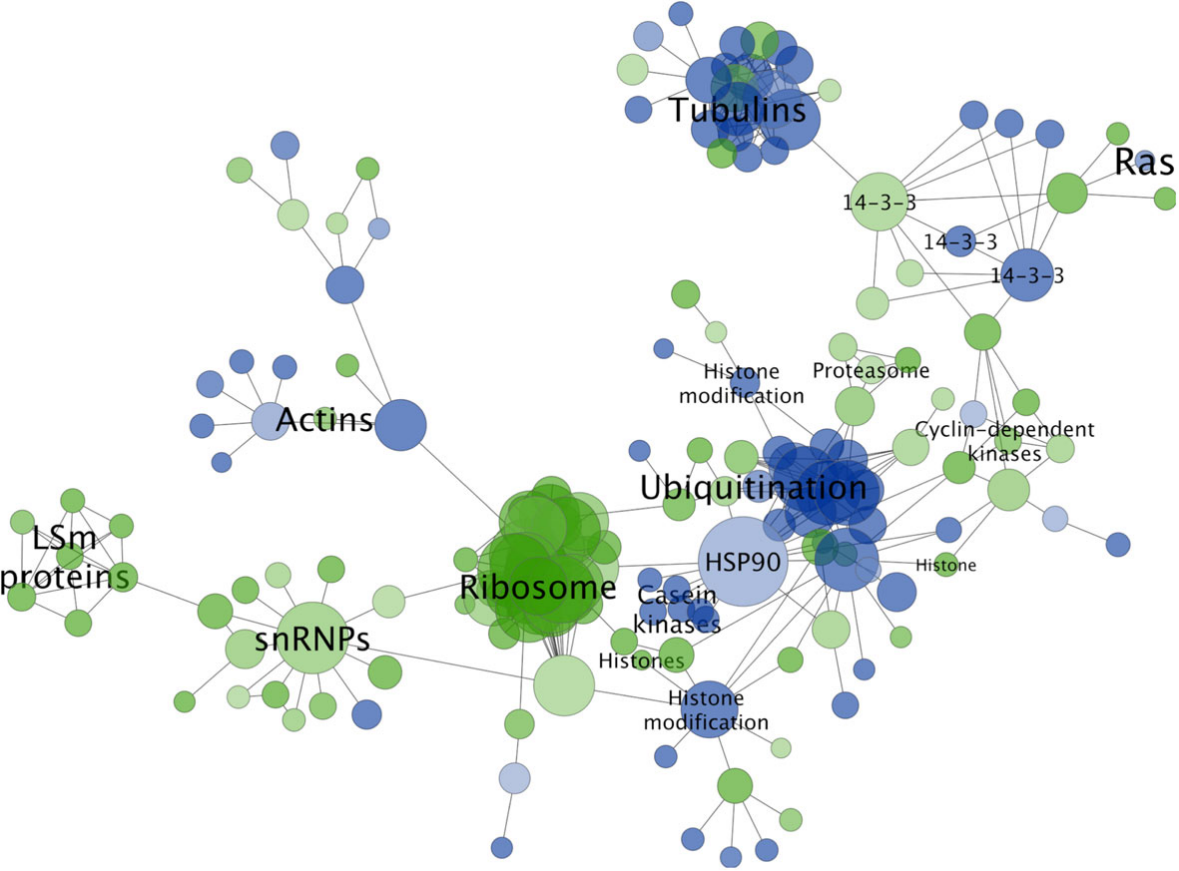
Differential expression. Differentially-expressed genes between adult and larval worms were mapped to Hm_HC_net and the sub-network extracted (green, up-regulated in larvae; blue, up-regulated in adults). Protein nodes are sized relative to the number of interactions they connect

We were interested in exploring interactomes specific to different life stages. We mapped differentially-expressed genes (DEGs) identified between adults and 5-day old, metamorphosing larvae [5] to the network to create a sub-network of these proteins (where the DEGs are present in the network). Of 3,479 DEGs, 367 were present in the network, and 176 proteins formed a connected component of 668 interactions (Fig. 14). There was little overlap between the DEGs and GOI interaction clusters (Figs. 11, 12 and 13) with the exception of tubulin beta-chains, which were mostly up-regulated in larvae, and cullin 1 proteins, which were up-regulated in adults (see Additional file 1 Table S2). Several members of the network clusters (Fig. 9) were represented in the DEG subnetwork, with protein production processes being up-regulated in larvae and cytoskeletal and ubiquitin-related processes up-regulated in adults. Of the hub proteins (Table 2) fourteen were differentially expressed (five up-regulated in adults and nine in larvae, see Additional file 1 Table S2).

In larvae, ribosomal and RNA splicing factors were significantly up-regulated. These proteins included a large ribosomal group corresponding to 23 of 27 proteins of cluster 1 in addition to twelve other ribosomal proteins. This ribosomal group was connected to a group of thirteen snRNP-related proteins that contained three proteins of cluster 4, which was, in turn, connected to the Lsm proteins of cluster 7. Four of the eight proteasomal subunits of cluster 2 were also up-regulated in larvae. A group of three histones are also up-regulated.

In adults, two groups of cytoskeletal proteins (actins and tubulins) were up-regulated, including seven members of cluster 5, one of which HmN_000742800, is un-annotated. A large connected group of ubiquitination-associated proteins were up-regulated in adult worms, with the exception of four proteins at the periphery of the group, which had higher expression in larvae. The heat shock protein HSP90, one of the largest hubs in Hm_HC_net with 52 interactions, was also up-regulated in addition to a group of 6 casein kinases, and two histone modification proteins.

Signalling proteins were split between up-regulation in larvae and up-regulation in adults. The majority of cyclin-dependent kinases, with the exception of one of the protein hubs (Table 2; Additional file 1 Table S2), present in the DEG network were up-regulated in larvae, while Ras was up-regulated in adult worms. Two 14-3-3 signalling proteins were up-regulated in adults and one in larvae. Interestingly, the three 14-3-3 proteins share interaction partners with one another. Full differential expression results are provided in Additional file 1 Table S2.

## Discussion

Although an effectively complete chromosome-level assembly and set of gene models are now available for *H. microstoma*, no system-level analysis has been conducted previously for the species. Hm_net provides the first steps toward using such data to understand tapeworm cellular biology by integrating interologs from sixteen eukaryotic species into an inferred interactome for *H. microstoma*. The network is probabilistic which reduces the impact of data set noise, in particular, from false positive interactions produced by high throughput studies, while assigning higher probabilities to interactions with multiple lines of experimental evidence [30]. Since the interologs are produced from a number of different species, these networks can have an additional level of noise, as not all interactions occurring in the source organisms will occur in *H. microstoma*. However, the confidence scoring schema also mitigates the effect of this noise in comparison to an unweighted interolog network [50]. We included a minimum count of 1.0 during the scoring stage in order to reduce loss of data in species with sparse interaction and gold standard data. While this would not be necessary or desirable in well-studied species, the thresholding of the network to remove low scoring interactions allows for the retention of those with multiple lines of low scoring evidence, which would otherwise have been lost without the minimum count.

The topologically-important nodes and clusters of Hm_net represent core housekeeping and essential processes, which is to be expected as these processes are common to all the species from which the interlogs are derived. For example, the COP9 signalosome is found in all eukaryotes [51] and subunits of this complex cluster together in Hm_net. Notably, patterns of differential expression correspond to the network clustering and are connected in areas of up- and down -regulation within the network. Therefore, the network connections provide a biologically-relevant picture of *H. microstoma* cellular biology.

It has been observed that interaction data, and annotations of proteins themselves, are biased towards certain biological processes such as protein biosynthesis and ribosomal proteins [52, 53], so it is unsurprising that Hm_net shows similar biases. The majority of significant proteins, clusters and differentially-expressed genes belong to essential, conserved processes, with the ribosomal proteins being prominent in all our assessment results. Some previous network-based studies have chosen to identify and remove these biases either during or following integration [54–56], and this approach may be of benefit when a less specific process is of interest. However, these approaches can come at the cost of the removal of valid and useful data [57].

One drawback of an interolog-base integration schema is the effect of redundant interologs which are based on the same evidence. *Hymenolepis microstoma* gene models with the same blast hits naturally have identical interactions and confidence scores in Hm_net. These interactions are likely to affect the results of some topological analyses, for instance, by artificially up-weighting the degree of some proteins and producing tighter clustering co-efficients. In several cases these interactions were apparent during clustering and subnetwork analysis, in particular the four Cullin 1 protein hubs (Table 2) and several clusters containing symmetrical redundant interactions, such as the posterior Hox proteins (Fig. 13). Redundancy is also likely to affect the identification of bottleneck proteins (that is, high betweeness centrality and low number of interactions). In some cases it may aid analysis by collapsing proteins and their interactions together if they have the same source evidence.

The major advantage of a network based approach is the ability to generate testable hypotheses for more focused experimental study in organisms lacking experimental data. It is noteworthy that many transcription factors are present in our networks, providing the potential to predict regulators and/or targets of genes of interest, which can be difficult to impossible from sequence analyses alone. In addition, of particular interest are the 14-3-3 proteins that feature prominently in Hm_net as a cluster of nine proteins, three of which are found in the differential expression subnetwork (two up-regulated in adult and one in larvae) and share interaction partners. These signalling ligands are highly conserved in eukaryotes [58] and are found in the excretory vesicles of the *Echinococcus granulosus* larvae where they may be used to modulate host immunity [59]. Focused study of these proteins and their shared interaction partners may aid in understanding host-parasite cross-talk [60].

Another protein of interest in Hm_net is Cdc14a (HmN_000237800), which has high betweenness but a relatively low number of interactions (21). This protein is involved in cell cycle arrest and is conserved between most of the species included in the interolog network build [61–63]. Cdc14a may represent a ‘bottleneck’ protein which is likely to be essential [40, 41]. Analysis of these and other network-based features has been used successfully in the prediction of essential genes across diverse organisms [64]. Prediction of synthetic lethal relationships between genes is another potential network use, for instance Benstead-Hume and colleagues used protein-protein interaction networks to predict human synthetic lethal interactions, which they then confirmed experimentally [65]. Such analyses may be used to identify targets for new chemotherapies in helminth research [66].

Networks may also be used to predict protein function based on interaction patterns, which is especially useful where there is no sequence similarity to other known proteins [67]. For example, HmN_000742700 and HmN_000742800, although un-annotated, cluster in the network with the tubulins (Fig. 9). Additionally, HmN_000742800 shares an expression profile with a large group of connected tubulin proteins (Fig. 14), making it a candidate for involvement on tubulin-related processes.

A potential use of this network is in comparative interactomics with other species in terms of presence/absence of interologs. Network comparison has the potential to identify areas of conservation and of divergence in interaction patterns [68]. The caveat to this type of approach is that the proteome of the comparison species must be as complete as that of *H. microstoma*, otherwise differences observed will be confounded by sampling error. However, both the human blood fluke *S. mansoni* and the tapeworm *E. multilocularis* have effectively complete proteomes, providing the potential for cross-species comparison. Protein network-based analysis will be key to understanding the interaction between parasite and host, and in identifying candidate drug targets to mitigate the disease burden of parasites [69, 70]. Several studies have made progress in this area [71–75], and cross-species comparisons at a systems-level, such as the probabilistic approach described here, will become a valuable tool in this area of research, particularly as new protein-protein interaction data accumulate.

We note that the network is far from complete in terms of proteome coverage (∼1/3 of proteins), but nevertheless covers a larger proportion of the somatic proteome than the equivalent network for the model worm *C. elegans*. In fact, the number of interactions for *C. elegans* is low, in comparison to other model species, which is likely due to there only being two HTP datasets available [76, 77]. The percentage of proteome coverage likely reflects this lack of *C. elegans* data combined with the evolutionary distance between *H. microstoma* and the other model species for which interaction data are available. In addition, we have concentrated on direct protein-protein interactions only. Inclusion of other types of interaction has the potential to increase this coverage of the *Hymenolepis microstoma* somatic proteome; for example, ‘regulog’ networks link orthologs of regulatory interactions [78] and ‘associalog’ networks link proteins/genes based on any type of interaction: physical, genetic, regulatory and other types of functional association [50, 79, 80]. However, these approaches generally come at the cost of introducing additional noise from false positive interactions [50, 79].

## Conclusion

Experimental demonstration of protein-protein interactions can require considerable effort and so far no high-throughput approach has been applied to parasitic flatworms. In a new study, Montagne and colleagues used a yeast two-hybrid system and additional means to investigate the downstream effectors of canonical Wnt signalling in tapeworms, showing that only one of three paralogs of *β*-catenin interacts with components of the canonical destruction pathway [81], similar to the situation in free-living planarians [82]. This represents one of the first such studies to test protein interactions in tapeworms, illustrating the scarcity of experimental data available for these important pathogens. With complete genomes now available, the application of systems level analyses can start to play an important role in ameliorating this deficit by consolidating knowledge derived from major model organisms. To help achieve this, in future studies we will expand Hm_net to include regulogs and associalogs, and perform comparative interactomics between *Hymenolepis microstoma* and other helminth species.

## Methods

### Network Integration

Networks were derived using a four-stage scoring, filtering, integration and thresholding method (Fig. 1a). Interaction data were downloaded from BioGRID^2^ (version 164, December 2018). BioGRID is a comprehensive and highly-curated resource for functional association data [29]. The database stores interactions of 28 different types, including both physical interactions (17 types), for instance from affinity capture and yeast two-hybrid studies, and genetic interaction evidence (11 types), such as dosage or synthetic growth defects. We filtered the data to remove non-eukaryotic and non-physical interaction types. Data were split into individual data sets by study and species, with low-throughput (LTP) studies (<200 interactions) grouped by experimental type (Additional file 1 Table S3), while high-throughput (HTP) studies (>=200 interactions) were treated as separate datasets (Additional file 1 Table S4). BioSystems pathways^3^ (version 20170421, downloaded 20^*th*^ February 2019) were used as the gold standard for confidence scoring. Confidence scores were calculated using the methods developed by Lee and colleagues [30], that calculates a log-likelihood score for each data set (1):

1 $$ lls^{L}(E) = \ln \left(\frac{P(L|E) /\neg P(L|E)} {P(L) /\neg P(L)} \right)  $$

where, *P*(*L*|*E*) and ¬*P*(*L*|*E*) represent the frequencies of linkages *L* observed in dataset *E* between genes annotated to the same and differing BioSystems pathways, respectively, and, *P*(*L*) and ¬*P*(*L*) represent the prior expectation of linkages between genes in the same and differing BioSystems pathways, respectively. Since interaction and gold standard data for some species were very sparse, a baseline count of one was used in all cases to ensure minimal loss of these datasets. A score greater than zero indicated that a dataset links genes annotated to the same pathway; higher scores indicate greater confidence in the predicted interactions. Datasets that did not have a positive score were discarded.

Orthologs of the *H. microstoma* proteome (version v.3) were identified with Blast+ (version 2.7.1) using the –gilist option to limit the search to NCBI identifiers from species in the BioGRID database (e-value <0.00001), and the results filtered for the top hit to BioGRID interacting proteins in each species. Identifier mappings were obtained from the UniProt [83] ftp server (downloaded 21 ^*st*^ February 2019). All *H. microstoma* splice variants were treated as single proteins to avoid redundant interactions. The BioGRID datasets were then filtered to retain interactions involving those proteins with orthologs (i.e. interologs), before being integrated using the Lee method [30] with a *D*-value of 1.0:

2 $$ WS = \sum\limits_{i=1}^{n} \frac {L_{i}}{D^{(i-1)}}  $$

where *L*_1_ is the highest confidence score and *L*_*n*_ the lowest confidence score of a set of *n* datasets.

For validating the predicted tapeworm interactome, networks for human, yeast and *C. elegans* proteomes were derived by integration of the unfiltered data sets specific to each of those species using the same integration pipeline. In this way, we compared the major parameters that describe the predicted Hm_net to those that describe networks based on empirical protein interaction data for the species.

### Network analysis

Network data were visualised in Cytoscape (version 3.7.1) [31]. Network statistics and plots were produced using the NetworkAnalyser plugin (version 2.7) [34]. Clustering was carried out using MCODE version 1.5.1 [84] with a degree threshold of 3, node score threshold of 0.2 and the ‘haircut’ option.

To examine if we could predict proteins that interact with specific genes of interest (Additional file 1 Table S5), we asked whether orthologs from four protein sets were present in the network, and where so, extracted the relevant sub-networks to examine the interologs:

1. Components of the Wnt, Notch and Hedgehog signalling pathways [4].
2. Hox family homeobox transcription factors [4].
3. Germline ‘multipotency’ genes [44].
4. Differentially-expressed genes with a log2 fold-change ≥2 between whole, gravid adults and 5-day old larval worms [5].

Differential expression was calculated by re-analysing previously available RNA-seq data [5] in order to take advantage of the more complete v.3 genome assembly and gene models. Briefly, raw reads were aligned to the genome using STAR [85] v2.4.2a with the *−alignIntronMin 10* option, count files were produced using featureCounts v1.6.3 [86], and differential expression assessed using DESeq2 [87] v1.20.0 with a *p*-value threshold of 0.00001. These results supercede those based on the v.2 genome in [5], and a full list of differentially-expressed genes among all of the RNA-seq samples generated in [5] is included in Olson (in preparation).

## Supplementary information

**Additional file 1** Tables S1-S5: **Table S1 Network clustering.** The proteins of the top ten clusters with in-cluster degree and MCODE protein score. Hub proteins (main text Table 2 are denoted *). **Table S2 Differential expression.** The 176 differentially-expressed genes identified between adults and 5-day old, metamorphosing larvae that form a sub-network of 668 interactions (main text Fig. 8). **Table S3 Low throughput data.** The low throughput (<200 interactions) datasets extracted from V164 of the BioGRID database. Interaction data were limited to physical interactions from eukaryotic species then split into individual datasets by study and species. Low throughput data were then grouped into datasets by BioGRID experimental type. **Table S4 High throughput data.** High throughput datasets extracted from V164 of the BioGRID database (>=200 interactions). Interaction data were limited to physical interactions (Ints) from eukaryotic species then split into individual datasets by species and by study based on PubMed ID (PMID). **Table S5 Genes of interest**. The protein components of signalling pathways, transcription factors and ‘multipotency’ germline/stem-cell-related genes used to extract subnetworks in main text section Specific genes of interest: signalling, transcription and germline-related genes.

**Additional file 2** Hm_net: Tab-delimited Hm_net dataset suitable for use with Cytoscape and other network viewers.

**Additional file 3** Hm_HC_net: Tab-delimited Hm_HC_net dataset suitable for use with Cytoscape and other network viewers.

**Additional file 4** Hm_net annotations. Tab-delimited Hm_net annotation dataset suitable for use with Cytoscape and other network viewers.

## Data Availability

The datasets integrated for this study can be downloaded at^4^ (version 164, December 2018) and 5 (version 20170421) from the BioGRID and BioSystems databases, respectively. Hm_net, Hm_HC_net and annotations are provided as a supplementary data file. *Hymenolepis microstoma* genome data are available from WormBase ParaSite^6^ (BioProject PRJEB124).

## Abbreviations

BC: Betweenness centrality
CC: Closeness centrality
CDC5L: Cell cycle division 5-like
DEG: Differentially expressed gene
FOX: Forkhead box
GOI: Genes of interest
HTP: High-throughput
LTP: Low-throughput

## Footnotes

1 https://parasite.wormbase.org/Hymenolepis_microstoma_prjeb124/

2 https://downloads.thebiogrid.org/BioGRID/Release-Archive/BIOGRID-3.5.168/

3 https://ftp.ncbi.nih.gov/pub/biosystems/biosystems.20170421/
