# Supplementary material for: The tapeworm interactome: inferring confidence scored protein-protein interactions from the proteome of Hymenolepis microstoma
Source: BMC Genomics. 2020 May 7;21:346. doi: 10.1186/s12864-020-6710-1 (PMC7204028; doi:10.1186/s12864-020-6710-1)
Supplement: Supplementary file 1 — Additional file 1 Tables S1-S5: Table S1 Network clustering. The proteins of the top ten clusters with in-cluster degree and MCODE protein score. Hub proteins (main text Table 2 are denoted *). Table S2 Differential expression. The 176 differentially-expressed genes identified between adults and 5-day old, metamorphosing larvae that form a sub-network of 668 interactions (main text Fig. 8). Table S3 Low throughput data. The low throughput (<200 interactions) datasets extracted from V164 of the BioGRID database. Interaction data were limited to physical interactions from eukaryotic species then split into individual datasets by study and species. Low throughput data were then grouped into datasets by BioGRID experimental type. Table S4 High throughput data. High throughput datasets extracted from V164 of the BioGRID database (>=200 interactions). Interaction data were limited to physical interactions (Ints) from eukaryotic species then split into individual datasets by species and by study based on PubMed ID (PMID). Table S5 Genes of interest. The protein components of signalling pathways, transcription factors and ‘multipotency’ germline/stem-cell-related genes used to extract subnetworks in main text section Specific genes of interest: signalling, transcription and germline-related genes. [file 12864_2020_6710_MOESM1_ESM.pdf]

Additional File 1 – Tables S1-S5

The tapeworm interactome: inferring confidence scored protein-protein interactions from the proteome of *Hymenolepis microstoma*

Katherine James [1,2,*], Peter D. Olson [2,*]
1 Department of Applied Sciences, Northumbria University, Newcastle upon Tyne, NE1 8ST.
2 Department of Life Sciences, Darwin Centre 1, The Natural History Museum, Cromwell Road, London SW7 5BD, United Kingdom
∗ katherine.p.m.james@northumbria.ac.uk and p.olson@nhm.ac.uk

Table S1: **Network clustering.** The proteins of the top ten clusters with in-cluster degree and MCODE protein score. Hub proteins (main text Table 2 are denoted *).

Cluster	Degree	Score	Gene model	Description
Cluster 1	25	22.847	HmN_000653200	40S ribosomal protein S12
Cluster 1	26*	23.778	HmN_000490400	60S ribosomal protein L4
Cluster 1	25	23.778	HmN_000390700	60S ribosomal protein L7a
Cluster 1	26*	23.778	HmN_003026960	40S ribosomal protein S8
Cluster 1	25	23.778	HmN_000102000	60S ribosomal protein L21
Cluster 1	26	23.778	HmN_000101000	60S ribosomal protein L18a
Cluster 1	25	22.847	HmN_000539200	60S ribosomal protein L19
Cluster 1	25	22.847	HmN_000257200	60S ribosomal protein L23a
Cluster 1	22	20.751	HmN_000817200	40S ribosomal protein S7
Cluster 1	26*	23.778	HmN_000077700	40S ribosomal protein S3a
Cluster 1	26*	23.778	HmN_000632900	40S ribosomal protein S23
Cluster 1	25	22.847	HmN_003033630	60S ribosomal protein L18
Cluster 1	25	22.847	HmN_003033620	60S ribosomal protein L18
Cluster 1	26	23.778	HmN_000384200	60S ribosomal protein L23
Cluster 1	26	23.778	HmN_003037990	60S ribosomal protein L37a
Cluster 1	26	23.778	HmN_003028820	60S ribosomal protein L5
Cluster 1	25	23.778	HmN_000306800	Proteasome subunit alpha type-3
Cluster 1	26	23.778	HmN_000211600	40S ribosomal protein S5
Cluster 1	25	23.778	HmN_000573200	60S ribosomal protein L24
Cluster 1	26*	23.778	HmN_000002900	40S ribosomal protein S4
Cluster 1	26	23.778	HmN_000021200	60S ribosomal protein L7
Cluster 1	26*	23.778	HmN_000899300	40S ribosomal protein S13
Cluster 1	26	23.778	HmN_000753600	40S ribosomal protein S11
Cluster 1	26*	23.778	HmN_000932000	40S ribosomal protein S16
Cluster 1	26	23.778	HmN_000646200	40S ribosomal protein S19
Cluster 1	25	22.847	HmN_000538600	60S ribosomal protein L19
Cluster 1	26*	23.778	HmN_000217500	40S ribosomal protein S2
Cluster 2	9	7.644	HmN_000759100	DNA-directed RNA polymerase II subunit RPB11
Cluster 2	12*	8.077	HmN_003006230	DNA-directed RNA polymerase II subunit RPB1

Continued on next page

Table S1: **Network clustering.** The proteins of the top ten clusters with in-cluster degree and MCODE protein score. Hub proteins (main text Table 2 are denoted *).

Cluster	Degree	Score	Gene model	Description
Cluster 2	11	7.152	HmN_000249300	DNA-directed RNA polymerases I, II, and III
Cluster 2	9	7.644	HmN_000692800	DNA-directed RNA polymerases I, II, and III
Cluster 2	11	7.152	HmN_000212300	DNA-directed RNA polymerases I, II, and III
Cluster 2	11	7.152	HmN_003041820	DNA-directed RNA polymerases I, II, and III
Cluster 2	9	7.644	HmN_000692700	DNA-directed RNA polymerases I, II, and III
Cluster 2	12	8.077	HmN_003043630	DNA-directed RNA polymerase II subunit RPB2
Cluster 2	9	7.644	HmN_000871800	DNA-directed RNA polymerase II subunit RPB9
Cluster 2	11	7.152	HmN_000254300	DNA-directed RNA polymerase II subunit RPB4
Cluster 2	12	8.077	HmN_000095200	Mediator of RNA polymerase II transcription subunit 10
Cluster 2	12	8.077	HmN_000546000	DNA-directed RNA polymerase II subunit RPB7
Cluster 2	12	8.077	HmN_000263100	DNA directed RNA polymerase II subunit RPB3
Cluster 3	7	6.000	HmN_000645700	Proteasome subunit beta type-2
Cluster 3	7	6.000	HmN_000417500	Proteasome subunit beta type-7
Cluster 3	7	6.000	HmN_003004800	Proteasome subunit alpha type-4
Cluster 3	7	6.000	HmN_000916400	Proteasome subunit beta type-3
Cluster 3	7	6.000	HmN_000681300	Proteasome subunit alpha type-2
Cluster 3	6	6.000	HmN_000813600	26S proteasome regulatory subunit 6B
Cluster 3	5	5.000	HmN_000969400	Proteasome maturation protein
Cluster 3	6	6.000	HmN_003000770	SWI/SNF-related matrix-associated actin-dependent regulator of chromatin subfamily A-like protein 1
Cluster 4	4	7.000	HmN_000362900	Pre-mRNA-splicing factor ISY1
Cluster 4	5	6.806	HmN_000566300	Pre-mRNA-splicing factor RBM22
Cluster 4	5	6.806	HmN_000845900	U5 small nuclear ribonucleoprotein 40 kDa protein
Cluster 4	8	7.418	HmN_000677000	Pre-mRNA-splicing factor SYF1
Cluster 4	6	6.533	HmN_000719900	Protein BUD31 homolog
Cluster 4	8	8.000	HmN_003036700	U5 small nuclear ribonucleoprotein 200 kDa helicase
Cluster 4	10*	7.418	HmN_002028700	U2 small nuclear ribonucleoprotein A'
Cluster 4	5	8.000	HmN_000463400	SNW domain-containing protein 1
Cluster 4	4	7.000	HmN_000879100	Small nuclear ribonucleoprotein E
Cluster 4	7*	8.000	HmN_000755100	Microfibrillar-associated protein 1
Cluster 4	10*	7.418	HmN_000632800	116 kDa U5 small nuclear ribonucleoprotein component
Cluster 5	5	4.691	HmN_003049000	Tubulin alpha-1B chain
Cluster 5	5	4.691	HmN_003048990	Tubulin alpha-1C chain
Cluster 5	8	4.667	HmN_000747100	Dynactin subunit 1
Cluster 5	8	4.022	HmN_000838500	Dystonin
Cluster 5	5	4.691	HmN_003038390	Tubulin alpha-1C chain
Cluster 5	5	4.691	HmN_003038410	Tubulin alpha-1C chain
Cluster 5	8	4.022	HmN_000742700	n/a

Continued on next page

Table S1: **Network clustering.** The proteins of the top ten clusters with in-cluster degree and MCODE protein score. Hub proteins (main text Table 2 are denoted *).

Cluster	Degree	Score	Gene model	Description
Cluster 5	5	4.691	HmN_003042330	Tubulin alpha-1C chain
Cluster 5	5	4.691	HmN_003038420	Tubulin alpha-1B chain
Cluster 5	8	4.022	HmN_000742800	n/a
Cluster 5	5	4.691	HmN_003038400	Tubulin alpha-1C chain
Cluster 5	8	4.022	HmN_000811700	ADP-ribosylation factor-like protein 2
Cluster 5	5	4.691	HmN_003049010	Tubulin alpha-1C chain
Cluster 6	5	5.000	HmN_000175400	Eukaryotic translation initiation factor 3 subunit I
Cluster 6	7	5.000	HmN_000616600	Eukaryotic translation initiation factor 3 subunit M
Cluster 6	3	5.067	HmN_003005280	COP9 signalosome complex subunit 5
Cluster 6	6	5.000	HmN_003044180	Eukaryotic translation initiation factor 3 subunit E
Cluster 6	5	5.000	HmN_000681800	Eukaryotic translation initiation factor 3 subunit D
Cluster 6	3	5.727	HmN_000167100	Cop9 signalosome complex subunit
Cluster 6	5	5.000	HmN_000273900	Eukaryotic translation initiation factor 3 subunit G
Cluster 6	3	5.067	HmN_000798600	COP9 signalosome complex subunit 2
Cluster 6	5	5.000	HmN_000665900	Eukaryotic translation initiation factor 3 subunit K
Cluster 7	4	4.000	HmN_000272300	U6 snRNA-associated Sm-like protein LSm5
Cluster 7	4	4.000	HmN_000888600	Probable small nuclear ribonucleoprotein G
Cluster 7	4	4.000	HmN_000138700	Probable U6 snRNA-associated Sm-like protein LSm4
Cluster 7	4	4.000	HmN_000842400	U6 snRNA-associated Sm-like protein LSm6
Cluster 7	4	4.000	HmN_000254100	U6 snRNA-associated Sm-like protein LSm3
Cluster 8	4	0.857	HmN_000070500	14-3-3 protein epsilon
Cluster 8	4	0.857	HmN_000209900	14-3-3 protein zeta/delta
Cluster 8	4	0.857	HmN_000780400	14-3-3 protein sigma
Cluster 8	4	0.857	HmN_000659700	14-3-3 protein beta:alpha
Cluster 8	5	1.048	HmN_003037390	Protein argonaute-2
Cluster 8	5	1.048	HmN_000551100	TBC domain-containing protein kinase-like protein
Cluster 8	5	1.048	HmN_000196600	Serine/threonine-protein kinase PknB
Cluster 8	4	0.857	HmN_000379400	14-3-3 protein zeta:delta
Cluster 8	5	1.048	HmN_000735000	Mitogen-activated protein kinase kinase kinase 1
Cluster 9	5	0.250	HmN_000309500	Cell division control protein 42 homolog
Cluster 9	5	0.222	HmN_000316900	Ras-related C3 botulinum toxin substrate 1
Cluster 9	4	0.222	HmN_000153000	Serine/threonine-protein kinase PAK 3
Cluster 9	5	0.250	HmN_003049740	Cell division control protein 42 homolog
Cluster 9	4	0.222	HmN_000447150	Serine/threonine-protein kinase PAK 3
Cluster 9	4	0.222	HmN_000059800	serine/threonine protein kinase PAK
Cluster 9	5	0.250	HmN_000337200	Cell division control protein 42 homolog
Cluster 9	4	0.200	HmN_000493200	serine/threonine protein kinase PAK
Cluster 9	4	0.222	HmN_000261000	Serine/threonine-protein kinase PAK 3

Continued on next page

Table S1: **Network clustering.** The proteins of the top ten clusters with in-cluster degree and MCODE protein score. Hub proteins (main text Table 2 are denoted *).

Cluster	Degree	Score	Gene model	Description
Cluster 10	5	0.222	HmN_000426100	Serine/threonine-protein kinase N1
Cluster 10	5	0.200	HmN_003040810	Protein kinase C beta type
Cluster 10	4	0.200	HmN_000173500	Transforming protein RhoA
Cluster 10	5	0.250	HmN_000671200	RAC-alpha serine/threonine-protein kinase
Cluster 10	5	0.222	HmN_000849600	Serine/threonine-protein kinase N2
Cluster 10	4	0.222	HmN_000530900	Receptor of activated protein C kinase 1
Cluster 10	4	0.200	HmN_000788000	Transforming protein RhoA
Cluster 10	4	0.200	HmN_000537500	Transforming protein RhoA
Cluster 10	4	0.250	HmN_003004910	Ras-related C3 botulinum toxin substrate 1

Table S2: **Differential expression.** The 176 differentially-expressed genes identified between adults and 5-day old, metamorphosing larvae that form a sub-network of 668 interactions (main text Figure 8). Hub proteins (main text Table 2 are denoted *).

Gene model	Degree	log2FoldChange	padj	Description
HmN_003048980	8	-12.84	3.14E-35	Tubulin beta-1 chain
HmN_003017900	31	-12.4	5.91E-234	Tubulin beta chain
HmN_000757600	2	-11.91	5.89E-29	F-box/WD repeat-containing protein 7
HmN_003000240	4	-11.79	4.86E-35	Tubulin alpha-3 chain
HmN_000221300	6	-11.24	6.49E-45	Tubulin alpha-4A chain
HmN_003049000	14	-11.22	9.34E-218	Tubulin alpha-1B chain
HmN_000300300	6	-11.2	2.83E-27	Casein kinase II subunit alpha
HmN_000994000	4	-10.9	9.35E-26	Ribosomal protein S6 kinase alpha-6
HmN_000139600	24	-9.65	2.63E-19	RING-box protein 1
HmN_000501400	16	-9.57	1.57E-19	E3 ubiquitin ligase complex SCF subunit sconC
HmN_003048860	24	-9.36	2.04E-53	Actin
HmN_003048990	12	-9.23	0	Tubulin alpha-1C chain
HmN_000483300	16	-9.22	2.44E-21	E3 ubiquitin ligase complex SCF subunit sconC
HmN_003044090	21	-9.13	1.34E-32	Tubulin beta-2B chain
HmN_003032460	6	-8.89	1.23E-20	Casein kinase II subunit alpha'
HmN_003036840	6	-8.84	7.61E-17	Casein kinase II subunit alpha'
HmN_003023870	6	-8.37	5.56E-51	Casein kinase II subunit alpha
HmN_003036830	6	-8.29	2.13E-24	Casein kinase II subunit alpha'
HmN_000063500	34*	-8.13	6.95E-36	Cullin-1
HmN_003006810	28	-8.04	2.11E-42	Histone acetyltransferase p300
HmN_002187600	4	-8.01	5.94E-14	Actin
HmN_003035160	26	-7.91	5.84E-222	Tubulin beta-4B chain
HmN_000145000	5	-7.73	5.17E-55	Cell division control protein 2 homolog
HmN_000341700	1	-7.45	6.68E-44	mRNA decay activator protein ZFP36
HmN_003049010	14	-6.61	7.33E-82	Tubulin alpha-1C chain
HmN_000259300	15	-6.56	1.85E-92	Serine/threonine-protein phosphatase PP1-gamma catalytic subunit
HmN_003038400	12	-6.55	1.92E-36	Tubulin alpha-1C chain
HmN_003003620	34*	-6.54	2.63E-117	Cullin-1
HmN_000145100	3	-6.4	5.35E-117	Cyclin-dependent kinase 9
HmN_003035350	7	-6.32	7.66E-07	Heat shock cognate 71 kDa protein
HmN_000180200	3	-5.91	2.50E-106	Peroxiredoxin-1
HmN_002145300	8	-5.49	1.57E-40	Histone-lysine N-methyltransferase SUV39H1
HmN_003035310	7	-5.25	1.05E-54	Heat shock cognate 71 kDa protein
HmN_000341500	9	-5.18	1.01E-67	14-3-3 protein gamma
HmN_000012700	6	-5.15	4.24E-51	General transcription factor IIE subunit 2
HmN_000629900	34*	-4.93	2.87E-12	Cullin-1

Continued on next page

Table S2: **Differential expression.** The 176 differentially-expressed genes identified between adults and 5-day old, metamorphosing larvae that form a sub-network of 668 interactions (main text Figure 8). Hub proteins (main text Table 2 are denoted *).

Gene model	Degree	log2FoldChange	padj	Description
HmN_003007120	14	-4.77	9.63E-10	DNA damage-binding protein 1
HmN_003012080	3	-4.72	4.98E-60	Peroxiredoxin-1
HmN_000376000	7	-4.62	3.49E-113	Tubulin alpha c chain
HmN_003018090	11	-4.58	1.88E-37	NEDD8-conjugating enzyme Ubc12
HmN_003018040	11	-4.52	4.50E-43	NEDD8-conjugating enzyme Ubc12
HmN_003038410	12	-4.47	1.10E-12	Tubulin alpha-1C chain
HmN_000742800	20	-4.12	8.95E-203	n/a
HmN_000135100	4	-4	3.55E-70	Actin
HmN_003035360	7	-3.68	1.03E-121	Heat shock cognate 71 kDa protein
HmN_000398500	2	-3.56	7.08E-283	Collagen alpha-2(I) chain
HmN_000906000	4	-3.41	2.54E-12	Tyrosine-protein kinase Yes
HmN_000144900	33*	-3.1	2.09E-59	Cyclin-dependent kinase 2
HmN_000780400	25	-3.1	2.71E-34	14-3-3 protein sigma
HmN_000768100	4	-2.88	5.99E-83	Actin
HmN_000826300	8	-2.75	1.17E-22	Probable ATP-dependent DNA helicase HFM1
HmN_000472100	7	-2.61	4.72E-52	Tubulin alpha chain
HmN_000112700	1	-2.41	1.46E-155	NEDD8-conjugating enzyme UBC12
HmN_000084600	2	-2.33	3.22E-115	Titin
HmN_003018120	10	-2.17	4.83E-06	NEDD8-conjugating enzyme Ubc12
HmN_000695800	14	-2.02	1.11E-19	Ras-related C3 botulinum toxin substrate 1
HmN_000214600	2	-1.93	4.65E-123	Glycogen [starch] synthase
HmN_003045210	6	-1.86	6.92E-45	Actin
HmN_003049740	7	-1.84	7.73E-47	Cell division control protein 42 homolog
HmN_000381000	22	-1.62	3.17E-91	Cullin-5
HmN_000410600	2	-1.6	1.47E-36	E3 ubiquitin-protein ligase TRIM56
HmN_003018190	10	-1.59	1.75E-16	NEDD8-conjugating enzyme Ubc12
HmN_000037600	1	-1.54	2.08E-35	Ras related protein ral-a
HmN_003028770	29	-1.5	9.78E-118	Tubulin beta-1 chain
HmN_003028760	8	-1.45	6.59E-21	Tubulin beta-1 chain
HmN_000781500	2	-1.42	2.19E-33	Triple functional domain protein
HmN_002036900	14	-1.24	9.72E-95	Transgelin-3
HmN_000490500	5	-1.21	8.71E-24	Ubiquitin-conjugating enzyme E2 H
HmN_000799800	52*	-1.14	2.21E-09	Heat shock protein HSP 90-alpha
HmN_003032900	5	-1.09	4.32E-47	Dynamin-1
HmN_000806400	6	-1.04	5.95E-12	Cyclin-dependent kinase 4
HmN_003040810	9	-1.02	8.19E-08	Protein kinase C beta type
HmN_003041270	3	1.01	2.42E-45	Tubulin beta-4B chain

Continued on next page

Table S2: **Differential expression.** The 176 differentially-expressed genes identified between adults and 5-day old, metamorphosing larvae that form a sub-network of 668 interactions (main text Figure 8). Hub proteins (main text Table 2 are denoted *).

Gene model	Degree	log2FoldChange	padj	Description
HmN_000884600	2	1.01	7.15E-08	SUMO-conjugating enzyme UBC9
HmN_003004280	9	1.02	6.81E-98	Tubulin alpha-1A chain
HmN_000306800	32	1.03	3.82E-09	Proteasome subunit alpha type-3
HmN_000417500	7	1.04	3.11E-38	Proteasome subunit beta type-7
HmN_000566300	10	1.04	1.56E-28	Pre-mRNA-splicing factor RBM22
HmN_000569700	3	1.04	2.28E-16	U2 small nuclear ribonucleoprotein B"
HmN_000493200	9	1.05	2.46E-12	Serine/threonine protein kinase pak
HmN_000694000	6	1.07	2.01E-25	Mitotic checkpoint protein BUB3
HmN_000123500	1	1.09	5.34E-11	Neurogenic differentiation factor 1
HmN_000538000	10	1.11	1.14E-56	26S proteasome regulatory subunit 4
HmN_003036340	31	1.12	2.65E-45	Elongation factor 1-alpha 1
HmN_000689500	3	1.12	4.15E-11	Transcription initiation factor TFIID subunit 9
HmN_000603600	2	1.15	6.14E-19	Triple functional domain protein
HmN_003003820	39*	1.18	3.57E-07	Eukaryotic initiation factor 4A-III
HmN_000209900	29	1.19	1.59E-61	14-3-3 protein zeta/delta
HmN_000840000	13	1.19	3.75E-09	Uncharacterized WD repeat-containing protein alr3466
HmN_000407100	5	1.23	6.95E-41	RNA helicase aquarius
HmN_000645700	7	1.24	8.32E-34	Proteasome subunit beta type-2
HmN_000806500	7	1.24	1.63E-26	Cyclin-Y-like protein 1
HmN_000109300	14	1.24	2.40E-25	Cyclin-dependent kinase 7
HmN_003028820	32	1.28	1.15E-107	60S ribosomal protein L5
HmN_000612100	8	1.3	1.50E-78	Small glutamine-rich tetratricopeptide repeat-containing protein alpha
HmN_000817200	27	1.31	1.47E-19	40S ribosomal protein S7
HmN_000569800	3	1.36	1.30E-53	U1 small nuclear ribonucleoprotein A
HmN_002028700	39*	1.37	2.39E-53	U2 small nuclear ribonucleoprotein A'
HmN_000520400	17	1.39	8.98E-17	Cyclin-dependent-like kinase 5
HmN_000262700	15	1.42	1.59E-38	Splicing factor 3A subunit 2
HmN_000490400	33*	1.43	5.98E-122	60S ribosomal protein L4
HmN_000539200	32	1.44	1.28E-93	60S ribosomal protein L19
HmN_003004800	15	1.52	3.83E-66	Proteasome subunit alpha type-4
HmN_003033620	29	1.52	3.89E-58	60S ribosomal protein L18
HmN_000077200	4	1.55	3.90E-109	E3 ubiquitin-protein ligase SIAH2
HmN_000005800	5	1.57	6.48E-25	p21-activated protein kinase-interacting protein 1
HmN_000759700	3	1.58	1.26E-111	Thioredoxin-dependent peroxide reductase
HmN_000052500	3	1.59	6.27E-11	Cofilin
HmN_003028780	21	1.61	4.17E-111	Tubulin beta-2B chain

Continued on next page

Table S2: **Differential expression.** The 176 differentially-expressed genes identified between adults and 5-day old, metamorphosing larvae that form a sub-network of 668 interactions (main text Figure 8). Hub proteins (main text Table 2 are denoted *).

Gene model	Degree	log2FoldChange	padj	Description
HmN_000077700	41*	1.66	2.78E-100	40S ribosomal protein S3a
HmN_002014200	11	1.68	1.09E-10	F-box/WD repeat-containing protein 7
HmN_000359200	6	1.7	7.53E-29	Cyclin-dependent kinase 14
HmN_000002900	37*	1.72	1.77E-180	40S ribosomal protein S4
HmN_000390700	31	1.73	4.63E-143	60S ribosomal protein L7a
HmN_000600900	4	1.73	2.52E-16	U6 snRNA-associated Sm-like protein LSm1
HmN_000403100	2	1.75	1.42E-99	Inosine-5'-monophosphate dehydrogenase 2
HmN_000573200	29	1.75	3.43E-40	60S ribosomal protein L24
HmN_000202800	6	1.77	9.67E-78	Small nuclear ribonucleoprotein-associated protein N
HmN_000257200	26	1.77	4.70E-28	60S ribosomal protein L23a
HmN_000530900	8	1.79	1.32E-247	Receptor of activated protein C kinase 1
HmN_003038390	14	1.83	4.06E-129	Tubulin alpha-1C chain
HmN_003026960	36*	1.89	1.86E-113	40S ribosomal protein S8
HmN_002125100	1	1.9	1.30E-76	40S ribosomal protein SA
HmN_000323900	10	1.93	8.08E-14	Ubiquitin-like protein 4A-A
HmN_000248600	12	2.05	1.26E-92	Mitogen-activated protein kinase 14
HmN_000217500	41*	2.24	4.40E-121	40S ribosomal protein S2
HmN_000050800	13	2.25	3.14E-09	Ubiquitin-conjugating enzyme E2 D1
HmN_000320300	11	2.36	8.84E-14	Peptidyl-prolyl cis-trans isomerase-like 1
HmN_003037560	3	2.4	2.39E-08	GTPase HRas
HmN_000062700	6	2.51	9.47E-11	Cyclin-dependent kinase-like 1
HmN_003044080	8	2.66	1.52E-101	Tubulin beta-2B chain
HmN_000849500	3	2.71	5.58E-55	E3 ubiquitin-protein ligase TRIM9
HmN_000101000	29	2.79	2.09E-35	60S ribosomal protein L18a
HmN_003037570	3	2.82	9.14E-08	GTPase HRas
HmN_000547500	1	2.87	2.37E-81	Histone H2A
HmN_000052600	3	2.98	5.73E-91	Actin-depolymerizing factor 1
HmN_000604400	4	3.06	6.53E-288	Late histone H1
HmN_000899300	35*	3.07	1.13E-43	40S ribosomal protein S13
HmN_000950100	15	3.08	5.00E-58	60S ribosomal protein L27
HmN_000578800	6	3.09	2.14E-40	U6 snRNA-associated Sm-like protein LSm8
HmN_000547600	12	3.18	3.07E-34	Histone H2B
HmN_002003300	2	3.27	3.41E-158	U1 small nuclear ribonucleoprotein C
HmN_000969400	5	3.3	8.52E-201	Proteasome maturation protein
HmN_000336400	11	3.3	1.63E-42	40S ribosomal protein S15
HmN_000545700	4	3.36	6.59E-86	40S ribosomal protein S30
HmN_000888600	4	3.59	1.07E-23	Probable small nuclear ribonucleoprotein G

Continued on next page

Table S2: **Differential expression.** The 176 differentially-expressed genes identified between adults and 5-day old, metamorphosing larvae that form a sub-network of 668 interactions (main text Figure 8). Hub proteins (main text Table 2 are denoted *).

Gene model	Degree	log2FoldChange	padj	Description
HmN_000102000	28	3.66	3.59E-143	60S ribosomal protein L21
HmN_000138700	6	3.78	2.08E-39	Probable U6 snRNA-associated Sm-like protein LSm4
HmN_000116700	5	3.83	3.08E-169	Protein transport protein Sec61 subunit beta
HmN_000735000	16	3.84	1.10E-155	Mitogen-activated protein kinase kinase kinase 1
HmN_000646200	31	3.84	2.23E-60	40S ribosomal protein S19
HmN_000812800	10	3.86	4.56E-53	Cyclin-dependent kinases regulatory subunit 2
HmN_000396000	6	4.05	4.46E-125	Small nuclear ribonucleoprotein Sm D2
HmN_000932000	37*	4.06	3.32E-253	40S ribosomal protein S16
HmN_000632900	35*	4.06	2.17E-117	40S ribosomal protein S23
HmN_000356900	18	4.3	6.87E-57	60S ribosomal protein L27a
HmN_000163800	5	4.36	8.40E-80	Activated RNA polymerase II transcriptional coactivator p15
HmN_000384200	31	4.37	3.84E-50	60S ribosomal protein L23
HmN_000102700	6	4.41	2.14E-100	Small nuclear ribonucleoprotein Sm D1
HmN_003035300	13	4.54	1.20E-222	Heat shock cognate 71 kDa protein
HmN_000652600	6	4.59	2.11E-112	Histone H2A
HmN_000068700	17	4.63	6.64E-163	60S acidic ribosomal protein P1
HmN_000753600	31	4.84	1.32E-303	40S ribosomal protein S11
HmN_000254100	7	5.05	2.86E-31	U6 snRNA-associated Sm-like protein LSm3
HmN_000653200	26	5.23	6.13E-121	40S ribosomal protein S12
HmN_000391400	6	5.48	1.03E-90	Small nuclear ribonucleoprotein Sm D3
HmN_000824700	9	5.52	1.69E-60	40S ribosomal protein S28
HmN_000842400	8	5.53	8.04E-23	U6 snRNA-associated Sm-like protein LSm6
HmN_002101000	17	5.54	4.78E-158	60S acidic ribosomal protein P1
HmN_000879100	12	5.58	1.17E-60	Small nuclear ribonucleoprotein E
HmN_000219600	15	5.66	3.33E-82	60S acidic ribosomal protein P2
HmN_000272300	4	5.88	4.05E-13	U6 snRNA-associated Sm-like protein LSm5
HmN_000070400	10	6.28	8.12E-178	40S ribosomal protein S17
HmN_000154900	7	6.31	2.18E-68	Small ubiquitin-related modifier 2
HmN_000236800	10	7.32	3.53E-86	40S ribosomal protein S21
HmN_003037990	30	8.59	6.62E-35	60S ribosomal protein L37a

Table S3: **Low throughput data.** The low throughput (< 200 interactions) datasets extracted from V164 of the BioGRID database. Interaction data were limited to physical interactions from eukaryotic species then split into individual datasets by study and species. Low throughput data were then grouped into datasets by BioGRID experimental type.

Species	Type	Interactions
Chlamydomonas reinhardtii	Affinity.Capture-MS	11
	Biochemical Activity	1
	Reconstituted Complex	2
Arabidopsis thaliana	Affinity Capture-Luminescence	46
	Affinity Capture-MS	1715
	Affinity Capture-RNA	21
	Affinity Capture-Western	1364
	Biochemical Activity	443
	Co-crystal Structure	45
	Co-fractionation	118
	Co-localization	58
	Co-purification	54
	Far Western	35
	FRET	569
	PCA	1168
	Protein-peptide	44
	Protein-RNA	8
	Reconstituted Complex	1682
	Two-hybrid	5020
Glycine max	Affinity Capture-Western	2
	Co-fractionation	1
	FRET	5
	Two-hybrid	21
Solanum lycopersicum	Affinity Capture-Western	2
	FRET	2
	Reconstituted Complex	10
	Two-hybrid	92
Solanum tuberosum	Two-hybrid	1
Zea mays	Affinity Capture-Western	2
	Biochemical Activity	2
	Co-fractionation	1
	PCA	1
	Reconstituted Complex	1
	Two-hybrid	2
Caenorhabditis elegans	Affinity Capture-MS	92
	Affinity Capture-Western	196

Continued on next page

Table S3: **Low throughput data.** The low throughput (< 200 interactions) datasets extracted from V164 of the BioGRID database. Interaction data were limited to physical interactions from eukaryotic species then split into individual datasets by study and species. Low throughput data were then grouped into datasets by BioGRID experimental type.

**Species**	**Type**	**Interactions**
	Biochemical Activity	19
	Co-crystal Structure	2
	Co-fractionation	4
	Co-localization	10
	Co-purification	3
	Far Western	13
	FRET	22
	PCA	4
	Protein-peptide	1
	Protein-RNA	3
	Reconstituted Complex	84
	Two-hybrid	458
Drosophila melanogaster	Affinity Capture-MS	2318
	Affinity Capture-RNA	472
	Affinity Capture-Western	4219
	Biochemical Activity	337
	Co-crystal Structure	71
	Co-fractionation	523
	Co-localization	96
	Co-purification	59
	Far Western	71
	FRET	74
	PCA	176
	Protein-peptide	6
	Protein-RNA	181
	Reconstituted Complex	381
	Two-hybrid	1081
Danio rerio	Affinity Capture-MS	1
	Affinity Capture-Western	51
	Biochemical Activity	4
	Co-crystal Structure	1
	PCA	1
	Reconstituted Complex	13
	Two-hybrid	81
Xenopus laevis	Affinity Capture-MS	62
	Affinity Capture-RNA	3

Continued on next page

Table S3: **Low throughput data.** The low throughput (< 200 interactions) datasets extracted from V164 of the BioGRID database. Interaction data were limited to physical interactions from eukaryotic species then split into individual datasets by study and species. Low throughput data were then grouped into datasets by BioGRID experimental type.

**Species**	**Type**	**Interactions**
	Affinity Capture-Western	207
	Biochemical Activity	41
	Co-crystal Structure	1
	Co-fractionation	23
	Co-localization	7
	Co-purification	1
	PCA	1
	Reconstituted Complex	75
	Two-hybrid	13
Gallus gallus	Affinity Capture-MS	243
	Affinity Capture-Western	69
	Biochemical Activity	3
	Co-crystal Structure	1
	Co-fractionation	3
	Co-localization	3
	Co-purification	4
	PCA	1
	Reconstituted Complex	30
	Two-hybrid	5
Macaca mulatta	Affinity Capture-Western	1
Homo sapiens	Affinity Capture-Luminescence	315
	Affinity Capture-MS	21501
	Affinity Capture-RNA	477
	Affinity Capture-Western	31023
	Biochemical Activity	6148
	Co-crystal Structure	845
	Co-fractionation	1038
	Co-localization	1645
	Co-purification	1263
	Far Western	602
	FRET	604
	PCA	513
	Protein-peptide	1522
	Protein-RNA	235
	Proximity Label-MS	660
	Reconstituted Complex	17913

Continued on next page

Table S3: **Low throughput data.** The low throughput (< 200 interactions) datasets extracted from V164 of the BioGRID database. Interaction data were limited to physical interactions from eukaryotic species then split into individual datasets by study and species. Low throughput data were then grouped into datasets by BioGRID experimental type.

**Species**	**Type**	**Interactions**
	Two-hybrid	10220
Canis lupus familiaris	Affinity Capture-MS	1
	Affinity Capture-Western	16
	Co-localization	2
	Reconstituted Complex	3
Sus scrofa	Affinity Capture-MS	32
	Affinity Capture-Western	12
	Co-crystal Structure	1
	Reconstituted Complex	3
	Two-hybrid	2
Bos taurus	Affinity Capture-MS	3
	Affinity Capture-Western	55
	Biochemical Activity	9
	Co-crystal Structure	2
	Co-fractionation	1
	Far Western	2
	Reconstituted Complex	17
	Two-hybrid	3
Ovis aries	Affinity Capture-Western	1
Oryctolagus cuniculus	Affinity Capture-Western	13
	Biochemical Activity	2
	PCA	1
	Reconstituted Complex	17
Cricetulus griseus	Affinity Capture-Western	1
Mus musculus	Affinity Capture-Luminescence	19
	Affinity Capture-MS	3469
	Affinity Capture-RNA	29
	Affinity Capture-Western	4807
	Biochemical Activity	372
	Co-crystal Structure	36
	Co-fractionation	195
	Co-localization	151
	Co-purification	41
	Far Western	64
	FRET	33
	PCA	12

Continued on next page

Table S3: **Low throughput data.** The low throughput (< 200 interactions) datasets extracted from V164 of the BioGRID database. Interaction data were limited to physical interactions from eukaryotic species then split into individual datasets by study and species. Low throughput data were then grouped into datasets by BioGRID experimental type.

**Species**	**Type**	**Interactions**
	Protein-peptide	151
	Protein-RNA	7
	Proximity Label-MS	55
	Reconstituted Complex	929
	Two-hybrid	845
Rattus norvegicus	Affinity Capture-Luminescence	4
	Affinity Capture-MS	801
	Affinity Capture-RNA	3
	Affinity Capture-Western	1061
	Biochemical Activity	96
	Co-crystal Structure	3
	Co-fractionation	61
	Co-localization	59
	Co-purification	12
	Far Western	13
	FRET	11
	PCA	3
	Protein-peptide	5
	Protein-RNA	2
	Reconstituted Complex	286
	Two-hybrid	219
Cavia porcellus	Affinity.Capture-Western	1
	Biochemical Activity	1
	Co-purification	1
	Reconstituted Complex	1
Vitis vinifera	FRET	1
Plasmodium falciparum 3D7	Co-crystal Structure	1
	Reconstituted Complex	1
	Two-hybrid	1
Oryza sativa Japonica Group	Affinity Capture-Western	2
	Biochemical Activity	1
	FRET	4
	PCA	2
	Reconstituted Complex	5
	Two-hybrid	26
Chlorocebus sabaeus	Affinity Capture-Western	4

Continued on next page

Table S3: **Low throughput data.** The low throughput (< 200 interactions) datasets extracted from V164 of the BioGRID database. Interaction data were limited to physical interactions from eukaryotic species then split into individual datasets by study and species. Low throughput data were then grouped into datasets by BioGRID experimental type.

**Species**	**Type**	**Interactions**
Selaginella moellendorffii	Reconstituted Complex	2
	Two-hybrid	4
Aspergillus nidulans FGSC A4	Affinity Capture-MS	49
	Affinity Capture-Western	5
	Biochemical Activity	1
	PCA	8
	Two-hybrid	1
Candida albicans SC5314	Affinity Capture-MS	400
	Affinity Capture-Western	122
	Biochemical Activity	24
	Co-crystal Structure	3
	Co-localization	3
	Co-purification	6
	Far Western	2
	PCA	4
	Protein-peptide	2
	Reconstituted Complex	14
	Two-hybrid	60
	Affinity Capture-Western	1
Schizosaccharomyces pombe 972h-	Affinity Capture-Luminescence	1
	Affinity Capture-MS	2514
	Affinity Capture-RNA	170
	Affinity Capture-Western	1364
	Biochemical Activity	240
	Co-crystal Structure	45
	Co-fractionation	52
	Co-localization	336
	Co-purification	204
	Far Western	27
	FRET	9
	PCA	8
	Protein-peptide	30
	Protein-RNA	9
	Proximity Label-MS	1
	Reconstituted Complex	469
	Two-hybrid	854

Continued on next page

Table S3: **Low throughput data.** The low throughput (< 200 interactions) datasets extracted from V164 of the BioGRID database. Interaction data were limited to physical interactions from eukaryotic species then split into individual datasets by study and species. Low throughput data were then grouped into datasets by BioGRID experimental type.

**Species**	**Type**	**Interactions**
Saccharomyces cerevisiae	Affinity Capture-Luminescence	34
	Affinity Capture-MS	12580
	Affinity Capture-RNA	734
	Affinity Capture-Western	9993
	Biochemical Activity	1892
	Co-crystal Structure	590
	Co-fractionation	878
	Co-localization	695
	Co-purification	1875
	Far Western	86
	FRET	166
	PCA	1327
	Protein-peptide	279
	Protein-RNA	296
	Proximity Label-MS	81
	Reconstituted Complex	5021
	Two-hybrid	4909

Table S4: **High throughput data.** High throughput datasets extracted from V164 of the BioGRID database (>=200 interactions). Interaction data were limited to physical interactions (Ints) from eukaryotic species then split into individual datasets by species and by study based on PubMed ID (PMID).

**Species**	**PMID**	**Ints**	**PMID**	**Ints**	**PMID**	**Ints**
Arabidopsis thaliana	18535787	200	23449474	396	21094157	536
	24846764	222	21734647	423	21189294	555
	19429840	246	24823379	469	23166809	666
	15805477	255	20855607	482	20706207	811
	22065421	276	24556609	506	23667124	941
	21423366	346	22737156	529	21798944	5506
	20407024	395	21952135	534	24833385	12051
Caenorhabditis elegans	18692475	688	14704431	4307		
Drosophila melanogaster	24114784	201	21447707	754	27626673	1800
	25453831	217	23749869	1217	22036573	10917
	22028469	386	15710747	1726	14605208	19886
	25242320	618	15575970	1759		
Xenopus laevis	26673895	637				
Homo sapiens	25192599	200	16196087	311	26687479	680
	24639526	201	21163940	311	15231748	692
	24244333	202	26777405	315	20562859	706
	25659154	203	23084401	317	17643375	709
	16159877	203	24797263	322	24255178	723
	15324660	208	24337577	323	16713569	764
	17314511	208	25147182	325	25281560	812
	22145905	208	23398456	326	20467437	853
	20186120	208	24344204	330	27621311	877
	19953087	211	25693804	331	22990118	947
	24705354	218	24623722	336	25963833	967
	24550385	219	22939624	363	23602568	984
	22493164	220	23824909	373	24332808	1008
	25756610	221	23752268	382	26673895	1037
	20020773	222	26167880	392	19738201	1068
	19295130	222	25324306	392	21516116	1125
	25277244	223	21319273	396	27880917	1321
	27637333	225	21903422	404	28611215	1488
	23349634	229	17620599	408	27432908	1510
	15175163	229	24189400	410	27499296	1514
	19060904	233	24999758	424	23443559	1514
	25515538	235	15231747	432	19615732	1538
	28378594	235	24366813	453	19322201	1669
	26641092	238	25036637	457	24711643	1736

Continued on next page

Table S4: **High throughput data.** High throughput datasets extracted from V164 of the BioGRID database (>=200 interactions). Interaction data were limited to physical interactions (Ints) from eukaryotic species then split into individual datasets by species and by study based on PubMed ID (PMID).

**Species**	**PMID**	**Ints**	**PMID**	**Ints**	**PMID**	**Ints**
	23125841	240	23463506	460	24457600	1776
	18782753	241	23455922	484	25609649	1822
	23246001	241	22623428	489	21832049	1893
	28190767	243	26831064	492	25921289	1921
	21182205	244	24722188	496	29128334	1975
	19156129	245	24778252	500	17353931	2050
	27926873	248	28561026	511	29117863	2296
	21081666	257	23455924	519	16169070	2449
	25737280	263	25468996	555	22658674	2473
	17043677	264	25241761	559	21900206	2604
	15604093	266	27591049	561	16189514	2605
	25315684	271	19549727	572	21988832	3433
	25900982	276	25640309	599	21145461	4540
	26514267	277	22365833	600	27173435	4905
	23667531	281	21044950	609	26638075	5347
	24163370	283	22268729	613	28431233	6219
	26389662	284	25814554	627	22863883	7129
	23383273	287	19380743	631	25416956	13424
	20811636	292	20936779	633	22939629	13917
	22119785	297	26618866	639	26496610	15215
	16055720	298	25852190	654	26344197	16795
	20133760	299	22586326	656	26186194	23709
	21244100	304	28065597	658	28514442	56467
	19690564	310	20211142	660		
Mus musculus	16944949	244	28190767	375	24457600	1163
	25737280	258	27507650	435	28378742	1664
	20362541	274	29459677	462	28671696	1994
	22922362	360	21565611	617		
	28706196	362	20211142	959		
Rattus norvegicus	26316108	214	25931508	354	22871113	729
Plasmodium falciparum 3D7	16267556	2435				
Schizosaccharomyces pombe 972h-	26167880	201	24013502	225	24713849	931
	22119525	202	22540037	298	26771498	1939
	21652630	225	21386897	347		
Saccharomyces cerevisiae	26804907	204	26217697	339	10688190	823
	15766533	205	21277287	352	21232131	933
	23209026	206	17922018	362	21460040	991

Continued on next page

Table S4: **High throughput data.** High throughput datasets extracted from V164 of the BioGRID database (>=200 interactions). Interaction data were limited to physical interactions (Ints) from eukaryotic species then split into individual datasets by species and by study based on PubMed ID (PMID).

Species	PMID	Ints	PMID	Ints	PMID	Ints
	22028667	216	12052880	370	23409723	995
	24374311	221	21825077	371	26493364	1080
	28539364	223	23911324	385	18805955	1089
	26777405	228	25172955	416	21734642	1122
	11743162	231	12150911	456	18757749	1206
	21926967	240	23712011	456	26668354	1244
	27550809	243	14660704	477	22271760	1320
	21944719	248	21107429	479	22940862	1412
	27905431	250	16606443	497	18719252	1610
	29361028	257	25792736	506	27863241	1686
	10900456	258	25228766	511	26524240	1751
	19106085	258	18087042	514	20489023	1849
	26392412	269	28096404	521	28768715	1875
	24500206	270	27552055	528	23222640	1886
	24152732	272	21558325	549	16093310	1941
	28325876	273	17634282	576	23332755	1973
	18046405	283	26849847	617	22199229	2007
	29738528	287	14759368	627	27821408	2053
	20595233	287	20170199	679	18467557	2395
	23945946	296	26831523	700	19840948	2541
	28539363	300	19536198	710	22875988	3207
	21070971	300	26673895	718	11805826	3210
	23403034	302	11283351	733	11805837	3598
	23831759	305	23904270	739	16319894	4021
	18667435	317	19841731	748	29158339	6390
	21386897	321	20508643	751	16429126	6531
	27373154	336	27580715	805	16554755	7075
	25452130	339	25026036	810		

Table S5: **Genes of interest**. The protein components of signalling pathways, transcription factors and multipotency germline/stem-cell-related genes used to extract subnetworks in main text section 3.4.

**Pathway**	**Name**	**Gene model**
Wnt/β-catenin canonical pathway	Wnt1	HmN_000328000
	Wnt2	HmN_000112200
	Wnt4	HmN_000808600
	Wnt5	HmN_000108100
	Wnt11a	HmN_002147900
	Wnt11b	HmN_000022800
	Fz5/8-FzA	HmN_000386300
	Fz4-FzB	HmN_000319700
	FzC	HmN_000494400
	Fz1/2/3/6/7-FzD	HmN_000227100
	FzE	HmN_000595200
	Dishevelled-A	HmN_000446600
	Dishevelled-B	HmN_000723000
	GSK3	HmN_000472700
	APC	HmN_000462000
	Axin	HmN_000572400
	β-catenin A	HmN_000161700
	β-catenin B	HmN_000192700
	LEF1/TCF	HmN_000448500
	WIF	HmN_000932100
	SFRP	HmN_000556500
	SFL	HmN_000359400
Ca2+ - dependent Wnt pathway	Phospholipase C	HmN_000108000
	Protein Kinase C	HmN_000418200
	Calcineurin	HmN_000416800
	CaMKII	HmN_000401300
Planar cell polarity Wnt pathway	Strabismus	HmN_000676800
	Prickle	HmN_000001700
	RhoA	HmN_000173500
	Rac	HmN_000316900
	ROCK (Rho-Kinase)	HmN_000273400
	JNK (JUN-Kinase)	HmN_000201000
Notch pathway	Notch1	HmN_000653600
	Notch2	HmN_000853800
	Numb	HmN_000511800
	HES1	HmN_000974100
	HES2	HmN_000343800
	HES3	HmN_000973500

Continued on next page

Table S5: **Genes of interest**. The protein components of signalling pathways, transcription factors and multipotency germline/stem-cell-related genes used to extract subnetworks in main text section 3.4.

Pathway	Name	Gene model
	Su(H)	HmN_000214100
	Dl1	HmN_000155700
	Dl2	HmN_000605500
	Dl3	HmN_000714400
	Dl4	HmN_000734400
	Dl5	HmN_000376400
	Dl6	HmN_000714300
	Pres	HmN_000031500
	Spdo	HmN_000234700
	Ofut	HmN_000485500
	Kuz	HmN_000907900
	TACE	HmN_000264000
	Nicastrin	HmN_000242800
	Pen2	HmN_000694400
	APH1	HmN_000691700
	SMRT	HmN_000838900
	Nle	HmN_000357200
Hedgehog pathway	Cubitus Interruptus	HmN_000840400
	Hedgehog	HmN_000068600
	Disp	HmN_000570400
	Patched	HmN_000602000
	Smoothened	HmN_000930900
	Su(fu)	HmN_000482400
	Fu	HmN_000686000
	Slimb	HmN_000834500
	PKA1	HmN_000251300
	PKA2	HmN_000254800
	CK1-1	HmN_003047480
	CK1-3	HmN_000690200
	Caesin Kinase -CK1-3	HmN_000690500
	CK1-4	HmN_000752200
	CK1-5	HmN_000297300
	CK1-6	HmN_000348900
	GSK3-B1	HmN_000354400
	GSK3-B2	HmN_000472700
	CBP-5	HmN_000756700
	CBP-7	HmN_000466400
	Rsp-1	HmN_000543900

Continued on next page

Table S5: **Genes of interest**. The protein components of signalling pathways, transcription factors and multipotency germline/stem-cell-related genes used to extract subnetworks in main text section 3.4.

**Pathway**	**Name**	**Gene model**
	Igu	HmN_000604900
	Rdx	HmN_000637800
	Ttv	HmN_000215200
	Sotv	HmN_000928900
	Botv	HmN_001006500
Hox transcription factors	Hox1	HmN_000092500
	Lox4/Abda	HmN_000400300
	Hox4/Dfd	HmN_000772500
	Lox5	HmN_000187700
	Post1a-1	HmN_003005880
	Post1a-2	HmN_003005900
	Post1b-1	HmN_003005850
	Post1b-2	HmN_003005860
	Post2	HmN_000187500
Stem-cell related	Ago1a	HmN_000114300
	Ago1b	HmN_000950000
	Ago2	HmN_000658200
	Bruno4	HmN_000599500
	DDX3	HmN_000061900
	DDXX	HmN_000797000
	efTu a	HmN_000017000
	efTu b	HmN_000074700
	klf10	HmN_000574300
	klf5a	HmN_000013000
	klf5b	HmN_000820000
	MCM4	HmN_000501500
	MCM5	HmN_000718100
	Nanos	HmN_000753700
	p63	HmN_000797200
	Pum1	HmN_000758500
	Pum2	HmN_000516200
	Pum3	HmN_000125500
	rbp4	HmN_000255100
	RNAhelME31B	HmN_000746800
	Zfp1	HmN_000668300
	Zfp2	HmN_000668400
	Sox-PF1	HmN_000208300
	Sox-PF2	HmN_000874900

Continued on next page

Table S5: **Genes of interest**. The protein components of signalling pathways, transcription factors and multipotency germline/stem-cell-related genes used to extract subnetworks in main text section 3.4.

**Pathway**	**Name**	**Gene model**
	Sox-PF3	HmN_000494900
